# FGFR signaling and neddylation facilitate SARS-CoV-2 infection by modulating interferon induction and viral entry, respectively

**DOI:** 10.1016/j.isci.2025.114566

**Published:** 2025-12-29

**Authors:** Alberto Felix-Lopez, Joaquin Lopez-Orozco, Mohamed Elaish, Nawell Fayad, Zaikun Xu, Tekeleselassie Woldemariam, Bardes B. Hassan, Rashmi Panigrahi, Juveriya Qamar Khan, Megha Rohamare, Irv Mayers, J.N. Mark Glover, Joyce A. Wilson, Darryl Falzarano, Anil Kumar, Tom C. Hobman

**Affiliations:** 1 Department of Medical Microbiology & Immunology, Faculty of Medicine & Dentistry, University of Alberta, Edmonton, AB, Canada; 2 Department of Cell Biology, Faculty of Medicine & Dentistry, University of Alberta, Edmonton, AB, Canada; 3 Poultry Diseases Department, Faculty of Veterinary Medicine, Cairo University, Giza, Egypt; 4 Department of Pathology, Faculty of Veterinary Medicine, Cairo University, Giza, Egypt; 5 Department of Biochemistry, Faculty of Medicine & Dentistry, University of Alberta, Edmonton, AB, Canada; 6 Department of Biochemistry, Memorial University of Newfoundland, St. John’s, NL, Canada; 7 Department of Biochemistry, Microbiology and Immunology, University of Saskatchewan, Saskatoon, SK, Canada; 8 Department of Medicine, University of Alberta, Edmonton, AB, Canada; 9 Vaccine and Infectious Disease Organization (VIDO), University of Saskatchewan, Saskatoon, SK, Canada; 10 Department of Veterinary Microbiology, University of Saskatchewan, Saskatoon, SK, Canada; 11 Li Ka Shing Institute of Virology, University of Alberta, Edmonton, AB, Canada

**Keywords:** Pharmacology, Natural sciences, Biological sciences, Microbiology, Virology

## Abstract

SARS-CoV-2 is the causative agent of COVID-19, and although vaccines have reduced disease severity, emerging variants remain a significant public health issue. Broadly effective therapeutics, particularly those targeting host pathways essential for coronavirus infection, are still needed. Here, we used a CRISPR knockout screen to identify druggable host factors required for SARS-CoV-2 infection. The screen revealed NAE1 and FGFR1 as key contributors to infection. Inhibitors, either FDA-approved or those in clinical trials, of these pathways reduced replication of both ancestral and contemporary viral variants. Mechanistic studies showed that FGFR1 promotes viral replication through downstream MEK/ERK signaling, while neddylation appears to support viral entry or infectivity rather than replication itself. In a murine model of severe COVID-19, inhibitors of NAE1 and FGFR1 significantly decreased viral load and lung pathology. These findings support the development of host-targeted antiviral strategies.

## Highlights

• NAE1 and FGFR1 are host dependency factors for SARS-CoV-2
• NAE1 activity is important at early stages of infection, likely viral entry
• FGFR1 and downstream signaling suppress interferon induction

## Graphical abstract

**Figure:**
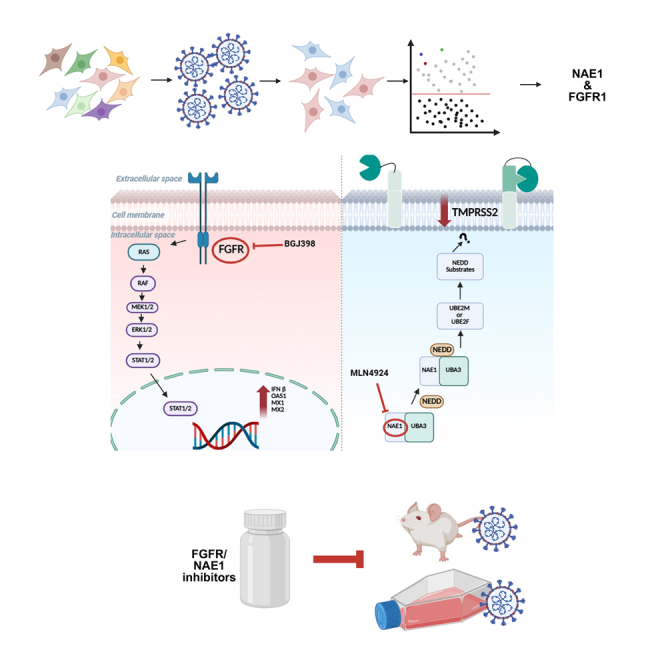

## Introduction

Severe acute respiratory syndrome coronavirus 2 (SARS-CoV-2), the causative agent of the COVID-19 pandemic, is the latest zoonotic coronavirus that has crossed over into the human population. The rapid development and deployment of vaccines greatly reduced the mortality from COVID-19, but the continual emergence of viral variants poses an ongoing challenge to vaccine efficacy. SARS-CoV-2 is now endemic in the human population, and reinfections are common in previously infected and vaccinated individuals.^1^^,^^2^ Several direct acting antivirals including viral polymerase (remdesivir and molnuparivir) and protease (paxlovid) inhibitors as well as monoclonal antibody therapies have been approved for treating COVID-19; however, their efficacies have been impacted with the emergence of new viral variants.^3^^,^^4^^,^^5^ As such, it is important to continue efforts to develop effective antivirals that reduce morbidity and mortality in patients with COVID-19.

As an alternative to small molecule inhibitor against viral targets, we and others have explored targeting host factors as therapeutic strategies to mitigate infection by SARS-CoV-2 and other coronaviruses. Like all viruses, SARS-CoV-2 engages many host proteins and cellular pathways during infection. Cellular proteins supporting virus replication are known as host dependency factors, and multiple genetic screens have identified human proteins that are important for SARS-CoV-2 infection including RAB7a,^6^ HMGB1,^7^ TMEM106B,^8^ and TMEM41B.^9^ To be useful as therapeutic targets, these host proteins must be able to be inhibited using small molecules. However, that is not the case for most of the SARS-CoV-2 host dependency factors identified so far.

To identify druggable host factors that are important for SARS-CoV-2 infection, we performed a genome-wide loss-of-function CRISPR-knockout screen in human embryonic kidney (HEK) 293T cells overexpressing the SARS-CoV-2 receptor angiotensin-converting enzyme 2 (ACE2). Top hits to which FDA-approved drugs or inhibitors in clinical trials were available were chosen for further study. We showed that SARS-CoV-2 infection is dependent upon the neddylation and fibroblast growth factor receptor (FGFR) pathways. Pharmacological inhibition of these pathways significantly reduced SARS-CoV-2 titers *in vitro* and in a mouse model of severe COVID-19.^10^^,^^11^ Pathway mapping and drug-resistance studies revealed that the inhibitors of Nedd8-activating enzyme 1 (NAE1) and FGFR1 block virus replication by distinct mechanisms. Specifically, FGFR1 inhibition enhances the interferon (IFN) response to viral infection, whereas blocking neddylation reduces levels of TMPRSS2 (transmembrane protease serine 2), which is needed for the cleavage of SARS-CoV-2 spike protein to initiate infection.^12^^,^^13^

## Results

### Novel host dependency factors for SARS-CoV-2

**Figure 1:**
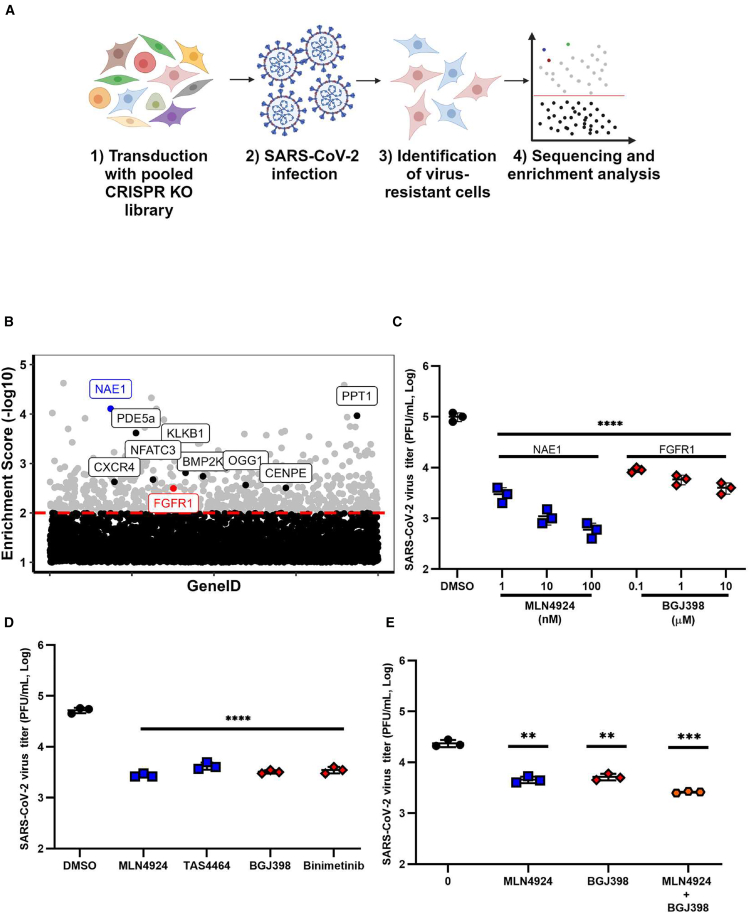
Identification of druggable host dependency factors for SARS-CoV-2 (A) Schematic of the pooled screen pipeline to identify SARS-CoV-2 host dependency factors in HEK293T-ACE2 cells. (B) Scatterplot showing the gene level enrichment score (-Log10) of disrupted genes in cells that are resistant to SARS-CoV-2 (Canada/ON/VIDO-01/2020) infection. The top ten druggable targets are outlined. The top druggable target is outlined in blue, and the tenth hit is outlined in red. (C) HEK293T-ACE2 cells were pretreated with inhibitors of the host factors for 24 h and then infected with SARS-CoV-2 (72B/CA/CALG), using an MOI of 0.1. Twenty-four hours later, the media was collected and subjected to plaque assay to determine viral titers. (D) HEK293T-ACE2 cells were pretreated with NAE1 inhibitors (TAS4464 and MLN4924) at 100 nM, FGFR inhibitor (BGJ398) at 10 μM, or MEK inhibitor (binimetinib) at 100 μM for 24 h and then infected with SARS-CoV-2 (omicron B.1.1.529 strain), using an MOI of 0.1. Twenty-four hours later, the media was collected and subjected to plaque assay to determine viral titers. (E) Primary normal human bronchial epithelial (NHBE) cells were pretreated with 1 μM MLN4924 or BGJ398 or both drugs (at 0.5 μM each) for 24 h and then infected with SARS-CoV-2 (72B/CA/CALG), using an MOI of 1. Twenty-four hours later, virus-containing media was collected and then subjected to plaque assay to determine viral titers. The average titers from three independent experiments are shown. Error bars represent the standard error of the mean. One-way ANOVA with Dunnett’s multiple comparison test was used to determine statistical significance between the control (DMSO) and drug-treated samples. *p* value < 0.01 ∗∗, <0.001 ∗∗∗, <0.0001 ∗∗∗∗ (C, D, and E).

To identify host dependency factors for SARS-CoV-2, we performed a gene knockout screen, using a human genome-wide pooled CRISPR guide RNA (gRNA) library. The Brunello library contains 76,441 gRNAs targeting a total of 19,114 genes with ∼4 gRNAs per gene.^14^ HEK293T cells stably over-expressing ACE2 (HEK293T-ACE2)^15^ were transduced with a lentivirus expressing Cas9 (CRISPR-associated protein 9). HEK293T-ACE2 cells were chosen for this screen because they are highly transducible and showed high cytopathic effect (CPE) when infected with SARS-CoV-2 (Figure S1). The resulting HEK293T-ACE2-Cas9 cell line was then transduced with the lentiviral gRNA library using a multiplicity of transduction (MOT) of 0.3, followed by selection with puromycin to generate a population of gene knockout (KO) cells.^16^ The KO cell pool was infected with a Wuhan-like strain of SARS-CoV-2 (Canada/ON/VIDO-01/2020) at an MOI of 5, and 72 h later, the genomic DNA from the surviving cells was extracted. The integrated gRNAs were PCR amplified and identified by deep sequencing. Three independent biological replicates were performed to generate a robust dataset. The distributions of the gRNAs in the infected and control samples were analyzed using the software Model-based Analysis of Genome-wide CRISPR-Cas9 Knockout (MAGeCK)^17^ (Figure 1A). The relative enrichments of the genes in infected samples were ranked to generate a candidate list (Figure 1B, Table 1).

**Table 1:** List of top-ranking druggable host targets from SARS-CoV-2 CRISPR KO screen

Rank	Druggable target	Inhibitors
6	NAE1	MLN4924
*9*	PPT1	DC661
*19*	PDE5a	Cpd7a
*101*	KLKB1	aprotinin
*120*	BMP2K	SGC-AAK1-1
*151*	NFAT	N7032
*164*	CXCR4	tannic acid
*190*	OGG1	OGG1 inhibitor O8
*223*	CENPE	GSK-923295
*228*	FGFR1	BGJ398

We compared our results with those of other host factor screens^6^^,^^7^^,^^9^^,^^18^^,^^19^ that used the same Brunello library with different cell lines as well as screens that also used HEK293T-ACE2 cells (Figure S2). When comparing the top 5% (1,000 hits) from each screen, we observed a high diversity in results. This can be explained in part by the differences in cell lines, infection times, MOIs, and viral strains. Validation studies were performed for the top 10 druggable targets from this list using their small molecule inhibitors, as shown in Figures 1C and S3.

Pharmacological inhibitors were used to study the effects of putative host dependency factors on SARS-CoV-2 (72B/CA/CALG) replication in HEK293T-ACE2 cells. Among the six drugs that showed significant, dose-dependent inhibitory effects on SARS-CoV-2 replication, four had a modest effect with <10-fold reduction at the highest noncytotoxic concentrations (Figures 1C and S3). Only drug concentrations that retained >90% cell viability were used for the antiviral assays (Figure S4). The drug MLN4924, which inhibits the top druggable target NAE1, reduced viral titers by more than 100-fold at the highest doses and showed a selectivity index (SI) of 5,626. BGJ398, which targets FGFR1, also inhibited the production of infectious SARS-CoV-2 (72B/CA/CALG) by more than 10-fold and showed an SI of 1,978 (Figure 1C) (Figure S5).

To determine whether the activities of NAE1 and FGFR1 are also important for the replication of more recent SARS-CoV-2 variants, we measured replication of the strain SARS-CoV-2 omicron B.1.1.529 in the presence of two inhibitors of NAE1 (MLN4924 and TAS4464), FGFR1 (BGJ398), and the downstream kinase MEK (binimetinib). All four of the inhibitors reduced omicron titers by over 90% (Figure 1D**),** suggesting that these are conserved host dependency factors among SARS-CoV-2 variants. To further confirm the importance of NAE1 and FGFR1 in SARS-CoV-2 replication and rule out the potential off-target effects of sgRNAs used in the original screen as well as drugs against these host dependency factors, clonal CRISPR KO cell lines lacking expression of these proteins were generated. Data in Figure S6 show that the knockout of NAE1 or FGFR1 reduced virus titers by more than 10-fold.

Next, we tested the importance of NAE1 and FGFR1 activity for SARS-CoV-2 (72B/CA/CALG) replication in primary normal human bronchial epithelial cells (NHBEs). In addition to being a physiologically relevant cell type for SARS-CoV-2 infection, it was important to rule out the possibility that the functions of NAE1 and FGFR1 as host dependency factors were not a cell type-specific phenomenon. NHBEs from three donors were pretreated for 24 h with the previously mentioned inhibitors and then infected with SARS-CoV-2; the viral titers were determined 48 h after infection (Figure 1E**)**. We measured the effects of various drugs or compounds on cell viability and used only the drug concentrations that retained >90% cell viability for antiviral assays (Figure S7). The NAE1 inhibitor MLN4924 and the FGFR inhibitor BGJ398 each reduced viral titers by more than 80%, and when used in combination, a modest but significant further reduction in viral titers was observed.

### Inhibition of FGFR enhances the IFN response

**Figure 2:**
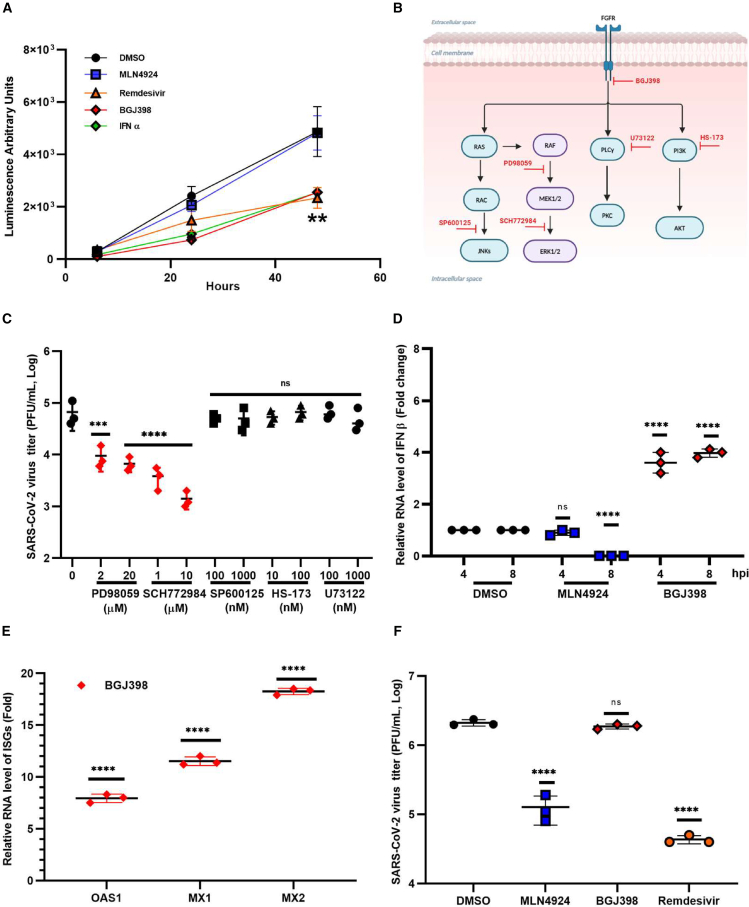
Inhibiting FGFR signaling enhances the interferon response (A) HEK293T-ACE2 cells were transfected with a SARS-CoV-2 nanoluciferase reporter replicon. Then, the cells were treated with MLN4924 (100 nM), BGJ398 (10 μM), remdesivir (10 μM), IFNα (100 unit/mL), or DMSO alone. Luminescence (expressed as arbitrary units) was measured at 24 and 48 h post-treatment. (B) Schematic of FGFR and downstream signaling pathways and inhibitors used to block them (indicated in red). (C) HEK293T-ACE2 cells were pretreated with the indicated inhibitors of signaling pathways downstream of FGFR for 24 h and then infected with SARS-CoV-2 (72B/CA/CALG) using an MOI of 0.1. Twenty-four hours later, media was collected and subjected to the plaque assay to determine viral titers. (D) Normal human bronchial epithelial (NHBE) cells were treated with DMSO alone, MLN4924, or BGJ398 at 1 μM for 24 h and then infected with 400 HAU/mL of Sendai virus. Total cellular RNA was harvested 4 or 8 h post infection (hpi) and then subjected to qRT-PCR to determine relative levels of mRNA encoding type I IFNβ. (E) NHBE cells were treated as described in (D). Total cellular RNA was harvested 8 hpi and then subjected to qRT-PCR to determine relative levels of mRNA encoding ISGs (OAS1, MX1, and MX2). (F) Vero cells were pretreated with MLN4924 (10 μM), BGJ398 (100 μM), remdesivir (10 μM), or DMSO for 24 h and then infected with SARS-CoV-2 (72B/CA/CALG), using an MOI of 0.01. Twenty-four hours later, the media was collected and subjected to the plaque assay to determine viral titers. Data shown are averaged from three independent experiments. Error bars represent standard error of the mean. One-way ANOVA with Dunnett’s multiple comparison test was used to determine statistical significance between the control (DMSO) and drug-treated samples. *p* value < 0.01 ∗∗, <0.001 ∗∗∗, <0.0001 ∗∗∗∗, ns = not significant (A, C, D, E, and F).

To determine if FGFR signaling was important for viral replication *per se*, we used a SARS-CoV-2 replicon to bypass the viral entry step. The replicon, which lacks the genes for spike and envelope proteins, expresses nano luciferase and neon green fluorescent protein reporters (Figure S8). Compared with DMSO alone, treatments with BGJ398, IFNα, and remdesivir reduced luciferase expression by as much as 60% (Figure 2A).

Next, we examined the potential mechanism of action of FGFR1 in SARS-CoV-2 replication. FGFR1 is a transmembrane receptor, tyrosine kinase, that functions in a wide variety of processes including cell survival, differentiation, proliferation, and immune cell trafficking and surveillance.^20^ FGFR1 signals through several downstream pathways including Ras, MAP kinase, phospholipase Cγ, and phosphatidylinositol 3-kinase (PI3K)/protein kinase B (AKT) that are known to affect replication of viruses.^21^^,^^22^ Multiple laboratories have confirmed that the Ras and MAP kinase pathways suppress the IFN response.^23^^,^^24^

To determine which signaling pathways downstream of FGFR1 are important for the replication of SARS-CoV-2 (72B/CA/CALG) (Figure 2B**)**, HEK293T-ACE2 cells were pretreated with inhibitors targeting key components of these pathways. Only drug concentrations that retained >90% cell viability were used for the antiviral assays (Figure S9). As seen in Figure 2C, drugs that inhibit the MEK/ERK pathway potently reduced SARS-CoV-2 replication in a dose-dependent manner. In contrast, inhibition of signaling through JNK or PI3K/AKT pathways did not measurably affect viral titers. Together, these data suggest that activation of the MEK/ERK pathway by FGFR1 and potentially other receptor tyrosine kinases is important for the replication of SARS-CoV-2.

Next, we assessed whether blocking FGFR activity affected the production of type I IFN and the induction of IFN-stimulated genes (ISGs) during viral infection of human primary lung epithelial cells. NHBE cells were pretreated for 24 h with BGJ398 and then infected with Sendai virus, a pathogen that causes a robust RIG-I-dependent IFN response.^25^^,^^26^^,^^27^ Total RNA was extracted 4 and 8 h post-infection, after which the relative levels of IFNβ, OAS1, MX1, and MX2 RNAs (Table 2) were determined by quantitative real-time PCR (qRT-PCR). Inhibiting FGFR activity resulted in significantly increased expression of IFNβ and ISG transcripts in response to viral infection (Figures 2D and 2E). Levels of secreted IFNβ were also significantly increased in BGJ398-treated samples (Figure S10). Conversely, levels of IFNβ RNA did not increase in Sendai virus-infected cells that were treated with the neddylation inhibitor MLN4924. Rather, at 8 h, there appeared to be a decrease in IFN induction (Figure 2D**)**. The reason for this is not known, but our observation is consistent with those of several previous studies.^28^^,^^29^^,^^30^

**Table 2:** Primers used for qRT-PCR

Target Gene	Primer sequences (5’→3′)
SARS-CoV-2 spike	fwd: CCTACTAAATTAAATGATCTCTGCTTTACT rev: CAAGCTATAACGCAGCCTGTA
*ACTB*	fwd: CCTGGCACCCAGCACAAT rev: GCCGATCCACACGGAGTACT
*ifnb*	fwd: TAGCACTGGCTGGAATGAGA rev: TCCTTGGCCTTCAGGTAATG
*Mx1*	fwd: GGCTGTTTACCAGACTCCGACA rev: CACAAAGCCTGGCAGCTCTCTA
*Mx2*	fwd: CAGCCACCACCAGGAAACA rev: TTCTGCTCGTACTGGCTGTACAG
*OAS1*	fwd: TTCTTAAAGCATGGGTAATTC rev: GAAGGCAGCTCACGAAAC

To further investigate the mechanism by which blocking FGFR signaling reduces SARS-CoV-2 replication, we pretreated Vero cells (which cannot produce type I IFN) with MLN4924, BGJ3988, remdesivir, or DMSO for 24 h prior to infection with SARS-CoV-2. Only drug concentrations that retained >90% cell viability were used for the antiviral assays (Figure S11). Data in Figure 2F show that unlike MLN4924 and remdesivir, which reduced viral titers by more than 10-fold, treatment of Vero cells with BGJ398 did not significantly affect the production of infectious virus. These data are consistent with a scenario in which FGFR signaling enhances SARS-CoV-2 replication by suppressing the IFN response.

### An internal deletion within the spike gene confers resistance to neddylation inhibitors

NAE1 is required for the first step in neddylation, a process that involves ligation of the ubiquitin-like molecule NEDD8 (neural precursor cell expressed developmentally downregulated protein 8) to lysine residues in substrate proteins.^29^ Many cellular proteins including Cullin family members, transcription factors, signal transducers, ribosomal proteins, and histones are regulated by neddylation.^29^^,^^31^^,^^32^ Of note, blocking NAE1 activity with MLN4924 has been shown to reduce the replication of multiple RNA and DNA viruses.^29^^,^^33^^,^^34^ This can occur by inhibiting the ability of viruses to co-op the neddylation pathway to target host restriction factors for degradation^35^ or by blocking neddylation of viral proteins that require this modification for stability and function.^36^

**Figure 3:**
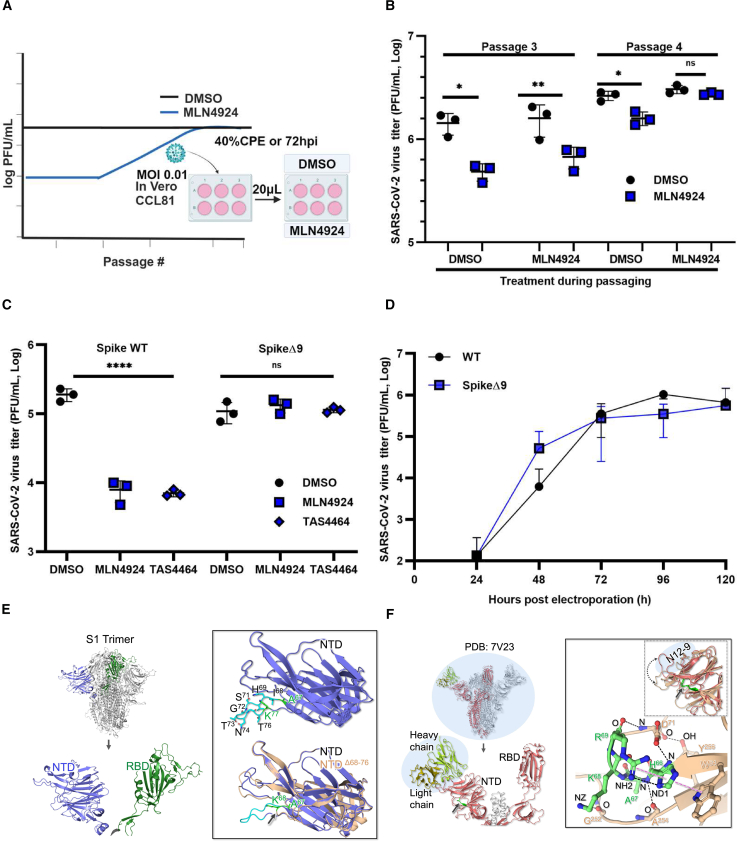
An internal deletion within the spike protein confers resistance to the neddylation inhibitor MLN4924 (A) SARS-CoV-2 (72B/CA/CALG) was passaged in Vero cells in the presence of MLN4924 or DMSO alone. For passage 1, an MOI of 0.01 was used for infection. Twenty microliters of media from infected cells were then used to infect a new batch of cells after 72 h or when 40% of the cells showed CPE. (B) Vero cells were pretreated with DMSO or MLN4924 (10 μM) for 24 h and then infected with aliquots of serially passaged SARS-CoV-2 (72B/CA/CALG) (MOI of 0.01) as explained in (A) for each passage. Twenty-four hours later, the media was collected and subjected to the plaque assay to determine viral titers. (C) HEK293T-ACE2 cells were pretreated with DMSO or MLN4924 or TAS4464 both at 100 nM for 24 h and then infected with SARS-CoV-2-WT or a SpikeΔ9 strain (MOI = 0.1). Twenty-four hours later, the media was collected and subjected to the plaque assay to determine viral titers. Data shown are averaged from three independent experiments. Error bars represent standard error of the mean. One-way ANOVA with Dunnett’s multiple comparison test was used to determine statistical significance between the control (DMSO) and drug-treated samples. *p* value < 0.05 ∗, <0.01 ∗∗, <0.0001 ∗∗∗∗, ns = not significant (B and C). (D) Titers from plaque assays conducted on media from Vero cells electroporated with infectious DNA clones for WT and SpikeΔ9. Error bars represent standard error of the mean. (E) Structural analysis of the spike protein deletion mutant NTD Δ68–76 associated with MLN4924 resistance. Left: mutant spike trimer model with one monomer highlighted (NTD in slate color, RBD in green). Right: NTD domains of wild type (slate) and the deletion mutant (wheat). In the wild-type spike, AlphaFold2 predicts that residues 68–76 (cyan sticks) form a disordered loop-like structure that is absent in cryo-EM structures. In NTDΔ68–76, the missing loop is indicated by an arrow. Residues A67 and K68 (corresponding to K77 in the NTD-WT) are shown in green. Despite the deletion, the overall NTD structure is preserved in the mutant. (F) Analysis of the spike NTDΔ68–76 bound to N12-9 Fab antibody (PDB: 7V23). The left image shows the spike trimer with one monomer (deep salmon) bound to the antibody. The antibody’s heavy and light chains bound to the NTD are colored in limon and deep olive, respectively. The right inset overlays the NTDΔ68–76 modeled using AlphaFold2 (wheat), with the NTDΔ68–76 bound to the antibody (PDB: 7V23, deep salmon). Antibody binding to the NTD induces movement of the Tyr248–Ser256 loop, as indicated by the dotted arrow. In the absence of the disordered loop (Δ68–76), the bordering residues His66, Ala67, Lys68, and Arg69 connect to form a more ordered structure as shown in green. This conformation is consistent between the cryo-EM and AlphaFold2 models. The green ball-and-stick representation in the bottom image highlights the network of interactions formed by these residues. Atoms involved in these interactions are labeled in black, with hydrogen bonds (black) and stacking interactions (pink) shown as dotted lines.

To explore the mechanism by which SARS-CoV-2 uses neddylation for infection, we serially passaged virus in Vero cells treated with or without MLN4924 to generate drug-resistant mutants (Figure 3A). SARS-CoV-2 remained sensitive to MLN4924 through the third passage, after which resistance was observed in the fourth passage (Figure 3B). RNA sequencing of viruses in passages 3 and 4 revealed a deletion of nucleotides 202 to 229 in the spike gene of two MLN4924-resistant isolates. This deletion results in a loss of nine amino acid residues (68–76) in the coding region for the N-terminal domain (NTD) in the extracellular region of the spike protein.^37^ No other mutations were detected in the drug-resistant viruses.

To confirm the link between MLN4924 resistance and the internal spike gene deletion, we used a novel reverse genetics system developed by the Ott laboratory^38^ to generate a molecular clone of SARS-CoV-2 (SpikeΔ9) with the same internal deletion in the spike gene (nucleotides 202 to 229) observed in the MLN4924-resistant viruses. A control wild-type (WT) isogenic strain of SARS-CoV-2 was also generated using the reverse genetic system. Data in Figure 3C show that the reconstituted SpikeΔ9 virus was resistant to MLN4924 and another NAE1 inhibitor TAS4464. Similar results were observed when the replication of SpikeΔ9 virus was assessed in NAE1 KO cells (Figure S12). The titers of the SpikeΔ9 virus and SARS-CoV-2-WT were similar in control (Cas9) cells, but in NAE1 KO cells, titers of WT SARS-CoV-2 titers were approximately 10-fold lower, whereas SpikeΔ9 virus titers were unaffected. These data suggest that the deletion of amino acids 68–76 in the NTD of the spike protein makes SARS-CoV-2 less reliant on neddylation in host cells. Finally, we analyzed replication kinetics by electroporating SARS-CoV-2-WT or SpikeΔ9 molecular clones at passage 0 to avoid compensatory mutations that may arise during passaging. We observed that at early timepoints, SpikeΔ9 titers were slightly higher than SARS-CoV-2-WT titers; however, at 72, 96, and 120 h postinfection, there were no differences (Figure 3D**)**.

Residues 68–76 of the NTD are absent from all the SARS-CoV-2 S1 cryo-electron microscopy (cryo-EM) structures in the PDB (https://www.rcsb.org/). Therefore, the NTD was modeled using AlphaFold2, which predicted that the residues 68–76 adopt a loop-like conformation. Given the absence of the corresponding density in the available cryo-EM structures, this prediction likely represents a plausible configuration of an intrinsically disordered and flexible region, rather than a well-defined structural element (Figure 3E**)**. The density for this loop is missing in cryo-EM structures likely due to its predicted flexibility. Modeling the MLN4924-resistant virus spike protein NTDΔ68–76 (SpikeΔ9) revealed that the bordering residues His66, Ala67, Lys68, and Arg69 form an ordered structure stabilized by a network of interactions. For example, cation-π interactions between Arg69 and His66 and π-stacking interactions between His66 and Trp64 were observed. Additionally, hydrogen bonding interactions occur between His66/Arg69, His66/Asp71, Ala67/Ala254, Lys68/Gly252, Arg69/Asp71, and Asp71/Tyr256. No other structural changes were observed between the NTD regions of Spike WT and the SpikeΔ9 mutant.

The modeled SpikeΔ9 structure is very similar to the PDB structure 7V23 (https://www.rcsb.org/structure/7V23); resolution: 2.9 Å), which is part of the prefusion complex that interacts with the fragment antigen-binding region of antibody N12-9 described in a study^39^ (Figure 3F). The conformations of SpikeΔ9 in the AlphaFold2 model and the PDB: 7V23 structure are consistent across all secondary structural elements. However, in the PDB: 7V23 structure, antibody binding to the NTD induces movement of the Tyr248-Ser256 loop (Figure 3F) but does not cause any structural changes in the NTD region where residues 68–76 have been removed. Our modeling suggests that the deletion of residues 68–76 in the NTD of SARS-CoV2 S1 does not disrupt the overall structure of this domain. Instead, the deletion promotes a more ordered configuration through a network of stabilizing interactions.

### The NAE1 inhibitor MLN4924 disrupts early stages of SARS-CoV-2 infection

**Figure 4:**
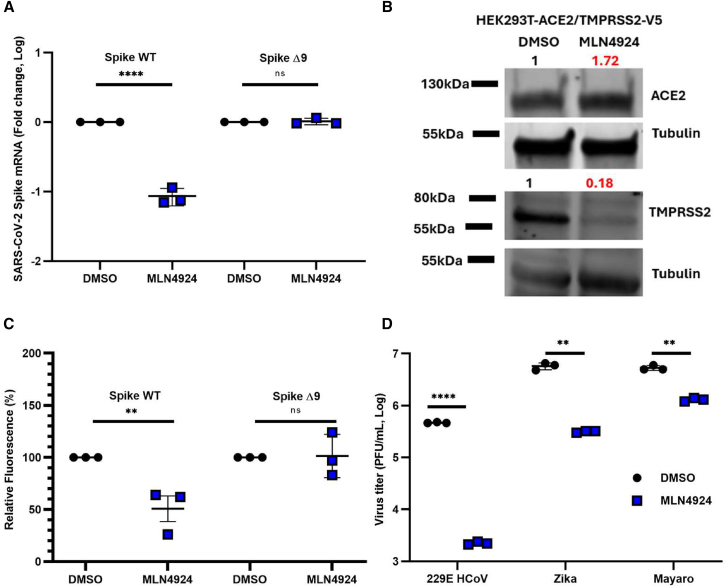
Neddylation is important for SARS-CoV-2 entry (A) HEK293T-ACE2 cells were pretreated with DMSO or MLN4924 (100 nM) for 24 h and then infected for 1 h with SARS-CoV-2-WT or SpikeΔ9 (MOI = 0.1), after which total RNA was extracted from the samples, and spike mRNA levels were determined by qRT-PCR. (B) Immunoblotting was used to assess the relative levels of ACE2 and TMPRSS2 protein in HEK293T-ACE2/TMPRSS2-V5 cells that were treated with DMSO or MLN4924 (100 nM) for 24 h. Numbers in red indicate intensity values normalized against respective control and tubulin. (C) GFP expression (based on total fluorescence per well) was measured in H23-ACE2 cells electroporated with Spike WT or SpikeΔ9 in the presence of DMSO or MLN4924 (10 nM). (D) At 24 h after treatment with DMSO or MLN4924, Huh7.5 or A549 cells were treated with MLN4924 (100 μM and 1 μM, respectively) or DMSO alone for 24 h and then infected with the indicated RNA viruses for 24 h. Viral titers of human coronavirus 229E (MOI = 0.5), Mayaro virus (MOI = 0.5), and Zika virus (MOI = 0.5) are shown. Error bars represent standard error of the mean. Paired Student’s *t* test was used to determine statistical significance between the control (DMSO) and drug-treated samples. *p* value < 0.01 ∗∗, <0.0001 ∗∗∗∗, ns = not significant (A, C, and D).

As seen in Figure 2A, unlike treatments with remdesivir, IFNα, or BGJ398, MLN4924 treatment had no effect on the propagation of SARS-CoV-2 replicon. To test if MLN4924 was affecting early steps of virus entry and/or replication, HEK293T-ACE2 cells were pretreated with MLN4924 or DMSO for 24 h, followed by infection with a WT strain of SARS-CoV-2 or the SpikeΔ9 mutant. Four hours later, the cells were harvested, and viral RNA was measured by qRT-PCR. MLN4924 treatment resulted in significantly lower levels of viral RNA in cells infected with SARS-CoV-2-WT but not in SpikeΔ9 virus-infected cells (Figure 4A).

Next, we investigated if MLN4924 had any effect on the replication of viral RNA when added post entry. HEK293T-ACE2 cells were infected with SARS-CoV-2-WT (MOI of 0.1) for 1 h, after which MLN4924 was added. We did not test SARS-CoV-2 SpikeΔ9 molecular clone since it is resistant to pretreatment with MLN4924. Media was harvested 24 h later, and viral titers were measured by the plaque assay. No significant differences were observed between DMSO- and MLN4924-treated samples (Figure S13), suggesting that NAE1 activity is important for viral entry. MLN4924 was also reported to inhibit early steps of alpha herpes virus entry.^40^ Since ACE2 and TMPRSS2 play important roles during SARS-CoV-2 virus attachment and entry into cells,^41^^,^^42^ we measured their expression levels in HEK293T-ACE2-TMPRSS2 cells. We observed a 90% reduction in TMPRSS2 protein levels in MLN4924-treated cells compared with control cells, whereas ACE2 protein levels were not affected by MLN4924 treatment (Figure 4B**)**.

The above findings are consistent with previous reports that the NTD of SARS-CoV-2 spike is important for TMPRSS2-dependant viral entry. Of note, SARS-CoV-2 variant spike proteins with a small deletion (ΔH69/V70) in the NTD domain mediate faster virus-cell fusion, a process that requires TMPRSS2, compared with spike proteins of WT strains.^43^^,^^44^^,^^45^ Furthermore, reports that swapped the NTD loops between SARS-CoV and SARS-CoV-2 S proteins found that SARS-CoV-2 with the shorter SARS-CoV loops are destabilized but more sensitive to proteolytic activation.^46^^,^^47^

Given the information above, we propose that SpikeΔ9 virus is less dependent on neddylation for entry into host cells because its structure facilitates enhanced proteolytic activation and therefore accelerated or enhanced efficiency of virus–cell fusion. To determine if MLN4924 affects spike protein-dependent syncytia formation, we used a split GFP (sGFP-N and sGFP-C) assay in cells expressing Spike WT or SpikeΔ9. TMPRSS2 activity is required for processing SARS-CoV-2 S protein to its fusogenic state, which is essential for syncytia formation.^45^ As such, monitoring syncytia formation can help assess TMPRSS2 presence or activity. H23-ACE2 cells were electroporated with plasmids encoding spike proteins and sGFP-N or sGFP-C. GFP fluorescence was measured following co-culture of the electroporated cells for 24 h. Reduced syncytia formation was observed in MLN4924-treated cells expressing Spike WT. In contrast, MLN4924 did not block syncytia formation in cells expressing SpikeΔ9 (Figure 4C**)**. Taken together, our results suggest that MLN4924 treatment reduces levels of TMPRSS2, which results in less activating cleavage of spike protein. Neddylation of TMPRSS2 has not been reported, and, thus, it is not clear if this post-translational modification affects its stability or not. However, multiple studies have shown that the NAE1 inhibitor MLN4924 can reduce the replication of other viruses that use TMPRSS2 48–50 including influenza virus and herpes simplex virus.^33^^,^^40^^,^^48^^,^^49^^,^^50^^,^^51^ Finally, we observed that MLN4924 inhibited the replication of other coronaviruses (SARS-CoV-2, SARS-CoV, and 229E) as well as RNA viruses such as Zika virus^35^ and Mayaro virus, which do not require TMPRSS2 for infection (Figures 4D, S14, andS15).

### Targeting host factors reduces viral load *in vivo*

**Figure 5:**
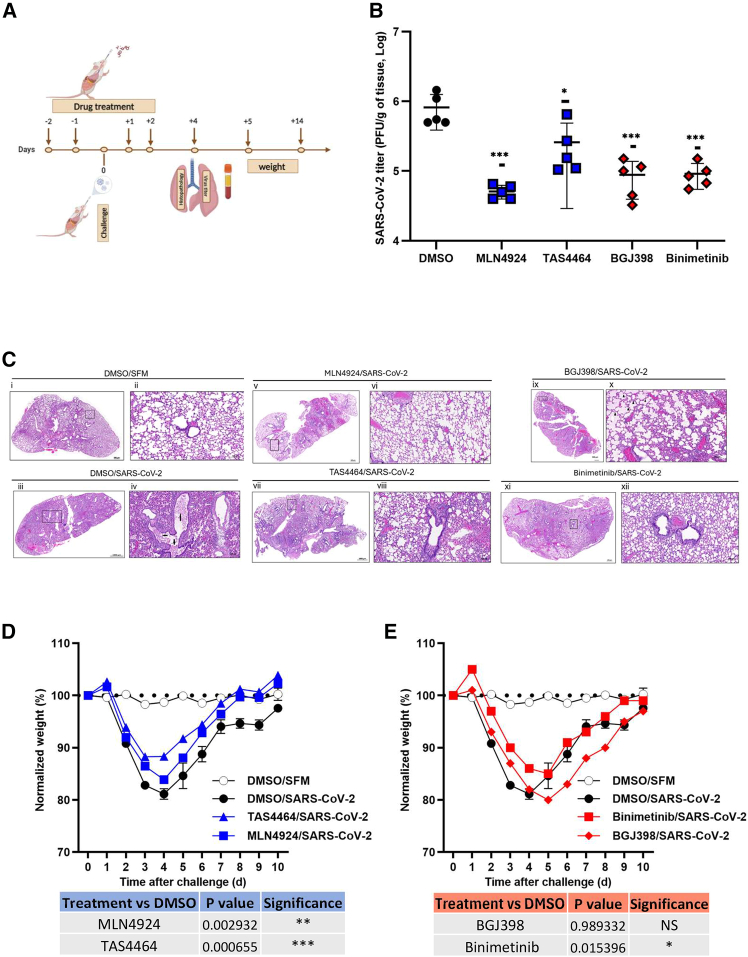
Targeting host factors reduces viral load and lung damage in mouse model for severe COVID-19 (A) Inhibitors of neddylation (MLN4924 and TAS4464) and FGFR signaling (BGJ398 and binimetinib) pathways were administered intranasally to female Balb/c mice once daily on days −2, −1, +1, and +2 relative to SARS-CoV-2 infection. One set of animals were humanely euthanized 4 days post-infection (dpi), and lung tissues were collected and processed for virus load (via plaque assay), viral RNA levels (via qRT-PCR), and histopathology analysis. Another set of animals were monitored daily for morbidity and weight for up to 14 days (B) Virus titers (pfu/g of tissue) from lungs of infected mice at 4 dpi (*n* = 5 mice per group) were determined by the plaque assay. Error bars represent standard errors of the mean. One-way ANOVA with Dunnett’s multiple comparison test was used to determine statistical significance between samples from the control (SFM/DMSO) and the mice treated with MLN4924 (15 mg/kg), TAS4464 (10 mg/kg), BGJ398 (10 mg/kg), or binimetinib (10 mg/kg). *p* value < 0.05 ∗, <0.001 ∗∗∗, <0.0001 ∗∗∗∗. (C) Histopathological analysis of mouse lung tissues. SFM/DMSO-treated uninfected (i, ii), SFM/DMSO-treated and infected (iii, iv), BGJ3398-treated and infected (v, vi), binimetinib-treated and infected (vii, viii), MLN4924-treated and infected (ix, x), and TAS4464-treated and infected (xi, xii). (ii), (iv), (vi), (viii), (x), and (xii) are enlargements of boxed areas in (i), (iii), (v), (vii), (ix), and (xi), respectively. Scale bars, 50 μm. SFM: serum-free media. (D and E) Weight changes in the infected mice treated with SFM/DMSO control, NAE1 inhibitors (MLN4924 and TAS4464), or FGFR1 inhibitors (BGJ398 and binimetinib) were plotted over the course of 14 days (*n* = 5 mice per group). Repeated-measures ANOVA was used to determine statistical significance between the control (DMSO) and drug-treated samples. *p* value < 0.05 ∗, ns = not significant.

To determine if targeting FGFR or NAE1 could reduce viral load and disease in a small animal model, we used a mouse-adapted strain of SARS-CoV-2 that causes severe disease in BALB/c mice.^10^ Vehicle alone or drugs that inhibit NAE1 or FGFR1 pathways were administered daily to mice by intranasal delivery two days before and two days after viral challenge, as described. Drugs were used at concentrations that did not significantly reduce the mice body weight (Figure S16). Four days post-infection, the mice were humanely euthanized, and viral load in the lungs was determined by plaque assay (Figure 5A). Animals treated with inhibitors of FGFR (BGJ398) and NAE1 (MLN4924 and TAS4464) had significantly lower viral loads in the lung, with MLN4924 having the most potent effect (Figure 5B). The MEK inhibitor binimetinib also reduced viral load in the lung by almost 10-fold, thus confirming the importance of MAP kinase signaling in SARS-CoV-2 replication.

To determine if any of the drugs reduced lung damage in infected animals, lungs from the uninfected and infected mice were fixed in formalin and then processed for histological examination using hematoxylin and eosin stain. Lung tissue sections were blind scored for pathology based on vascular involvement (incidence of congestion, incidence of hemorrhage, or perivascular cuffing), alveolar involvement (thickening of alveolar septa), and bronchiolar involvement (peribronchial infiltrate, hyperplasia of the bronchiolar epithelium, or sloughing of the bronchiolar epithelium).

Lungs from non-infected vehicle-treated mice showed normal pulmonary parenchymal appearance, whereas infected animals that did not receive drugs exhibited typical broncho interstitial viral pneumonia including thickening of the alveolar septae due to congestion and dilation of alveolar blood vessels (asterisks), immune cell infiltration, and hyperplasia of type II pneumocytes, leading to alveolar collapse (Figure 5C). Damage to lungs in BGJ398-, binimetinib-, MLN4924-, and TAS4464-treated mice was much less severe. Specifically, thickening of alveolar septae, bronchiolar walls, and blood vessel wall was markedly reduced (black arrows).

A parallel group of vehicle- and drug-treated infected animals were monitored for 14 days post-infection, and body weights were recorded daily. Infected mice that were treated with the NAE1 inhibitors MLN4924 and TAS4464 lost the least amount of weight and recovered quicker than vehicle-treated infected mice. The MEK inhibitor (binimetinib) also reduced weight loss in infected mice as expected; however, inhibition of FGFR with BGJ398 did not prevent weight loss in mice, even though it showed a similar effect on viral load reduction as binimetinib (Figures 5D and 5E). It is possible that the lack of protection from weight loss was due to side effects of the drug that may have affected eating and/or drinking.

## Discussion

High-throughput genetic screening technologies have enabled rapid identification of host factors that play functional roles in the SARS-CoV-2 life cycle. Unfortunately, most of the host targets identified to date are not readily targeted with clinically approved drugs, and the mechanisms by which they affect virus replication remain poorly characterized.^6^^,^^7^^,^^8^^,^^9^ Here, we report the identification and in-depth characterization of two druggable host cell pathways (NAE1 and FGFR signaling) that significantly impact SARS-CoV-2 infection *in vitro* and *in vivo*. Interestingly, NAE1 and FGFR1 were not identified as dependency factors for SARS-CoV-2 by previous CRISPR screens that used the same Brunello gRNA library.^6^^,^^8^^,^^18^^,^^52^^,^^53^ The reason for this is unclear but may be related to the differences in cell lines used in those screens. NAE1 and FGFR1 were important for infection by an early ancestral Wuhan strain as well as more recent omicron variants, indicating that neddylation and FGFR signaling are critical host processes for this coronavirus. Mapping studies showed that signaling through MEK/ERK is the most important pathway downstream of FGFR for SARS-CoV-2 replication. The reduced virus replication observed in FGFR1-depleted or drug-treated cells can be partially explained by the enhanced upregulation of type I IFNs and ISGs (OAS1,^54^ MX1,^55^ and MX2). This finding is in line with our previous observation that FGF signaling through the ERK-MEK pathway negatively regulates the IFN pathway, promoting the replication of another RNA virus, Zika virus.^22^ A subsequent study on herpes simplex virus 1 (HSV-1) and lymphocytic choriomeningitis virus confirmed out findings that FGF signaling facilitates virus replication by antagonizing the interferon pathway.^56^ Finally, the observation that high levels of FGF in sera are associated with adverse outcomes in COVID-19 patients^57^^,^^58^ provides yet more robust evidence that the FGFR signaling pathway is critical for SARS-CoV-2 infection.

While FGFR and downstream kinases are important for suppressing the innate immune response to SARS-CoV-2, the neddylation pathway appears to support viral entry. The requirement for NAE1 activity is indirect as we were unable to detect neddylation of spike protein or any other SARS-CoV-2 proteins by mass spectrometry (data not shown). Inhibition of neddylation using the NAE1 inhibitor MLN4924 caused a marked reduction in ectopically expressed TMPRSS2-V5. While this experimental system is not ideal, it enabled robust tracking of TMPRSS2 levels through its V5 tag. Future investigations should assess its endogenous expression and stability. TMPRSS2 is a cytoplasmic membrane cellular protease important for activating the fusion activity of spike protein. Indeed, blocking NAE1 activity resulted in decreased spike-dependent syncytia formation. Levels of the SARS-CoV-2 receptor ACE2, were not affected by MLN4924 treatment. A nine amino acid deletion in the N-terminal region of spike protein was sufficient to confer resistance to MLN4924. The ability of this mutant spike protein (SpikeΔ9) to induce syncytia was not inhibited by MLN4924. It remains to be determined how this deletion reduces the dependence on neddylation, but it is important to point out that the SpikeΔ9 mutant is similar to a number of SARS-CoV-2 variant spike proteins that exhibit enhanced viral entry.^43^^,^^44^ Further characterization of this mutant will yield valuable insights into the mechanisms of spike cleavage and viral membrane fusion.

We also found that MLN4924 reduces infection of other pathogenic RNA viruses including HCoV-229E (*Coronaviridae*), SARS-CoV, Zika virus (*Flaviviridae*), and Mayaro virus (*Togaviridae*), which supports the idea that targeting the neddylation pathway should be further investigated as a broad-spectrum antiviral strategy. Indeed, other investigators have shown that NAE1 inhibitors inhibit multiple DNA and RNA viruses including human cytomegalovirus,^59^ herpes simplex virus 1,^33^ hepatitis B virus,^36^^,^^60^ influenza A virus,^34^ Rift Valley fever virus^61^ and infectious pancreatic necrosis virus.^62^ . While the mechanisms underlying the sensitivity of all of these different viruses to MLN4924 and other neddylation inhibitors remain poorly understood, it is clear that MLN4924 either functions as a direct-acting antiviral (hepatitis B virus^36^) or targets host factors that modulate viral entry (SARS-CoV-2 [this study]) or replication (Zika virus^35^).

Both NAE1 and FGFR inhibitors reduced viral load and lung damage in a mouse model of severe COVID-19. Moreover, given that some FGFR inhibitors^63^ are clinically approved for use in humans and advanced clinical trials are ongoing for NAE1 inhibitors,^64^ it is conceivable that such drugs could be rapidly repurposed as antivirals against emerging coronaviruses and potentially other viral pathogens. With several small molecules targeting host factors of SARS-CoV-2 in clinical trials (reviewed in^65^), host factors-based approaches emerge as a viable strategy for developing broad-spectrum antivirals. Combining direct-acting antivirals that target viral proteins with those against host factors could lead to broad-spectrum antiviral strategies with improved efficacy and reduced chance for the emergence of resistance. For RNA viruses in particular, the high rate of mutations during genome amplification can lead to the rapid emergence of mutants that are resistant to drugs that target viral proteins. Hence, strategies combining host-based therapies that target multiple host dependency factors may offer a more durable therapeutic strategy. However, our findings with the NAE1 inhibitor MLN4924 offer a note of caution. While the genetic barrier to resistance for host-targeted antivirals is often touted as an advantage over direct-acting antivirals, the fact that we were able to isolate MLN4924-resistant SARS-CoV-2 mutants after as few as four passages suggests that this is not always the case. Whether other viruses can rapidly acquire resistance to this drug is not yet known.

Finally, the fact that inhibitors of NAE1 and FGFR1 pathways had a synergistic effect against SARS-CoV-2 bodes well for using combinations of host-targeted antivirals as well as combining host-targeted antivirals with direct-acting antiviral drugs as a strategy to effectively suppress viral infection and minimize the appearance of drug-resistant mutants from endemic and emerging viral pathogens.

### Limitations of the study

Although the current study utilized a CRISPR KO screen in HEK293T cells stably expressing human ACE2, the findings from this screen may differ from those in other models. The screen employed the SARS-CoV-2 D614G variant of the Wuhan ancestral strain, and although some of the main findings were validated using more contemporary omicron variant, we did not extend these studies to all other known SARS-CoV-2 variants. This study does not explore the influence or the association of sex, gender, or both on the results. Additionally, the *in vivo* experiments were performed using a genetically engineered mouse-adapted SARS-CoV-2 strain. Finally, the mechanism by which neddylation affects TMPRSS2 or other surface proteases stability needs further investigation. Despite these limitations, this study offers meaningful insights into antiviral drug repurposing and underscores the therapeutic potential of targeting proviral host factors.

## Resource availability

### Lead contact

Further information and requests for resources and reagents should be directed to and will be fulfilled by the lead contact, Dr. Tom C. Hobman (thobman@ualberta.ca).

### Materials availability

Newly generated materials are available from the corresponding authors upon reasonable request.

### Data and code availability

The datasets generated during this study are provided in the supplementary tables. The raw sequencing data are available at NCBI (GEO: GSE307418). This study does not report any original code. Any additional information required to reanalyze the data reported in this paper is available from the lead contact upon request.

## Acknowledgments

This work was supported by grants to T.C.H., A.K., and J.L.-O. from the 10.13039/100022992Canadian Institutes of Health Research (GA1-177707) and to T.C.H. from Alberta Ministry of Technology and Innovation through the SPP-ARC (Striving for Pandemic Preparedness - The Alberta Research Consortium) and the 10.13039/501100000196Canada Foundation for Innovation. VIDO acknowledges operational support from the 10.13039/501100000196Canadian Foundation for Innovation through the Major Science Initiatives program and from the Government of Saskatchewan through 10.13039/100015147Innovation Saskatchewan. The work in A.K. lab is funded by CIHR grants PUU-177963, PTT-179810, and MM1-181121. The work in J.A.W and D.F lab is funded by CIHR Operating COVID-19 Rapid Research Funding Opportunity-Therapeutics (VR3-172626). The authors thank V. Mancinelli and E. Reklow for technical support and Angela Johnson and Amy May for coordinating the collection of bronchoscopy samples. SARS-CoV-2 infection experiments were performed at the 10.13039/100006516University of Alberta's Containment Level 3 Facility, which receives administrative support from the 10.13039/501100017001Li Ka Shing Institute of Virology. SARS-CoV infection experiments were performed at VIDO, 10.13039/100008920University of Saskatchewan.

## Author contributions

A.F.-L., J.L.-O., A.K., and T.C.H. designed and managed the overall project. A.F.-L. drafted the original manuscript. A.F.-L., J.L.-O., and N.F. performed and analyzed *in vitro* experiments. A.F.-L., M.E., and Z.X. performed and analyzed *in vivo* experiments. B.B.H. analyzed the histopathology data. R.P. and J.N.M.G. performed the structural analysis of spike proteins. T.W. and D.F. performed and analyzed the SARS-CoV assays. J.Q.K. performed and analyzed IC_50_ experiments. M.R. and J.W. provided SARS-CoV-2 replicon. I.M. provided the normal human bronchial epithelial cells. All authors approved the final version to be published.

## Declaration of interests

The authors declare no competing interests.

## STAR★Methods

### Key resources table

**Table:** 

REAGENT or RESOURCE	SOURCE	IDENTIFIER
**Antibodies**		

V5 Tag Monoclonal Antibody	ThermoFisher	Cat# R960-25; RRID: AB_2556564
ACE2 antibody	Abcam	[EPR4435(2)]; RRID AB_2861381
Goat anti-rabbit conjugated to Alexa 680	Life Technologies	Cat#A21076; RRID: AB_2535732
Donkey anti-rabbit conjugated to Dylight 800	Life Technologies	Cat# SA5-10044; RRID: AB_2556624
Goat anti-mouse conjugated to Alexa 750	Life Technologies	Cat# A-21037; RRID: AB_2535708
Nedd8 antibody	Cell Signaling	Cat# 2745S; RRID: AB_10695300
b-Actin antibody	Sigma Aldrich	Cat# A3853; RRID: AB_262137
b-Tubulin antibody	Cell Signaling	Cat# 2146; RRID: AB_2210545

**Bacterial and virus strains**		

SARS-CoV-2 (Canada/ON/VIDO-01/2020)	GISAID	EPI_ISL_425177
Omicron B1.1.529	Dr. Lorne Tyrell (University of Alberta)	N/A
72B/CA/CALG	Dr. John Conly (University of Calgary)	Xu et al.^10^
Mouse adapted SARS-CoV-2	Generated in Hobman lab (University of Alberta)	Xu et al.^10^
Wuhan variant SARS-CoV-2 with D614G mutation in Spike	Vaccine and Infectious Disease Organization, Saskatoon, SK	N/A
Sendai Virus	Charles River laboratories	Ca#10100774
Mayaro virus	Scott Weaver (University of Texas Medical Branch)	strain 07-18066-99
Zika virus	Dr. Mike Diamond (Washington University School of Medicine)	N/A
SARS-CoV	Vaccine and Infectious Disease Organization, Saskatoon, SK	N/A
HCoV-229E	ATCC	VR-740
SARS-CoV-2 WA1	Generated in Hobman lab (University of Alberta)	Described in *Taha* et al.^38^
SARS-CoV-2 WA1 SpikeΔ9	Generated in Hobman lab (University of Alberta)	N/A

**Chemicals, peptides, and recombinant proteins**		

BGJ398	Sellekchem	Catalog#S2183
MLN4924	Sellekchem	Catalog#S7109
Tannic Acid	Sellekchem	Catalog#S3951
Binimetinib	Sellekchem	Catalog#S7007
TAS4464	Sellekchem	Catalog#S8849
PD98059	Sellekchem	Catalog#S1177
SCH772984	Sellekchem	Catalog#S7101
HS-173	Sellekchem	Catalog#S7356
SP600125	Sellekchem	Catalog#S1460
DC661	Sellekchem	Catalog#S8808
GSK923295	Sellekchem	Catalog#S7090
Aprotinin	Sellekchem	Catalog#S7377
U73122	Sellekchem	Catalog#S8011
Remdesivir	Sellekchem	Catalog# S8932
Cpd7a	Milipore Sigma	Catalog#508957
NFAT inhibitor	Milipore Sigma	Catalog#N7032
OGG1 inhibitor O8	Milipore Sigma	Catalog#SML1697
SGC-AAK1-1	TOCRIS	Catalog#6528
IFNα	Abcam	ab48750

**Deposited data**		

Raw and analyzed data	This paper	Geo: GSE307418

**Experimental models: Cell lines**		

Vero	ATCC	CCL-81
HEK 293T	ATCC	CRL-1573
HEK293FT	ThermoFisher	R70007
Vero E6	ATCC	CRL-1586
Huh 7.5	Tyrrell lab	N/A
A549	ATCC	CCL-185
Calu-3	ATCC	HTB-55
Vero-TMPRSS2	JCRB	1819
HEK293T-ACE2	Generated in Hobman lab (University of Alberta)	N/A
NCI-H23-ACE2	Generated in Hobman lab (University of Alberta)	N/A
C6/36	ATCC	CRL-1660
NHBEs	(Dr. Irv Mayers, Dept Medicine, U of Alberta)	University of Alberta human ethics protocol Pro00099685

**Experimental models: Organisms/strains**		

Balb/c	Charles River Laboratories	N/A

**Oligonucleotides**		

Primers used for RT-qPCR, Table 2	N/A	N/A

**Recombinant DNA**		

Brunello Library	AddGene	Cat# 52963
SARS-CoV-2 Replicon	Synthesized by Telesis Bio (formerly Codex DNA). Joint project between Kumar and Wilson labs.	N/A
TMPRSS2-V5	Human TRC 3.0 arrayed ORF library. MilliporeSigma	Clone ID: TRCN0000467270
FGFR1-V5	Human TRC 3.0 arrayed ORF library. MilliporeSigma	Clone ID: TRCN0000468162
NAE1-V5	Human TRC 3.0 arrayed ORF library. MilliporeSigma	Clone ID: TRCN0000470601
Spike WT	AddGene	Cat# 177960
SpikeΔ9	This paper	N/A
NAE1 sgRNA	AddGene	Cat# 90777
FGFR1 sgRNA	AddGene	Cat# 76067
sGFP-C (pBiFC-bFosVC155)	AddGene	Cat# 22013
sGFP-N (pBiFC-bJunVN155(I152L))	AddGene	Cat# 27098
pcDNA™5/TO	ThermoFisher	V103320

**Software and algorithms**		

MAGeCK v0.5.9.5	N/A	https://anaconda.org/bioconda/mageck
Graphpad Prism 8	N/A	N/A
Biorender	N/A	N/A
R-studio	N/A	N/A
Snapgene	N/A	N/A
ColabFold v1.5.5	N/A	N/A
AlphaFold2	N/A	N/A
AlphaFold3	N/A	N/A
ImageJ	N/A	N/A
CLC Genomics Workbench 20 software	N/A	N/A
Odyssey Image Studio Lite software	N/A	N/A

### Experimental model and subject details

#### Cell lines

Vero CCL81, HEK 293T, HEK293FT, Vero E6, Huh 7.5, A549 and Calu-3 cells were sourced from the American Type Culture Collection (Manassas, VA), Vero-TMPRSS2 from JCRB cell bank 1819, and HEK 293T-ACE2 and NCI-H23-ACE2 were developed in-house by stable transduction of ACE2 expressing lentivirus constructs. All cell lines except H23-ACE2 cells were cultured in Dulbecco’s modified Eagle’s medium (DMEM) (Gibco) supplemented with 100 U/ml penicillin and streptomycin, 1 mM HEPES (Gibco), 2 mM glutamine (Gibco), 10% heat-inactivated fetal bovine serum (FBS) at 37°C in 5% CO_2_. H23-ACE2 were grown in RPMI 1640 medium (Gibco) supplemented with 100 U/ml penicillin and streptomycin, 1 mM HEPES (Gibco), 2 mM glutamine (Gibco), 10% heat-inactivated fetal bovine serum (FBS) at 37°C in 5% CO2. Primary normal human bronchial epithelial (NHBE) cells obtained from bronchoscopy patients (University of Alberta human ethics protocol Pro00099685) were cultured in BEGM Bronchial Epithelial Cell Growth Medium (Lonza, cat. no. CC-3170) in a humidified atmosphere at 37°C and 5% CO_2_. Experiments carried out on NHBE cells were performed using cells from three donors. Donors were allocated at random. Cells lines were not authenticated. Cells were routinely tested for mycoplasma contamination.

### Method details

#### Virus stocks

SARS-CoV-2 infections were carried out in the Containment Level 3 Facility at the University of Alberta (Edmonton, Canada) using SARS-CoV-2 virus (Canada/ON/VIDO-01/2020; GISAID accession no. EPI_ISL_425177). Clinical isolation representing Omicron B1.1.529 variants of SARS-CoV-2 was kindly provided by Dr. Lorne Tyrrell (University of Alberta). A clinical isolate of the early D614G variant (72B/CA/CALG) was provided by Dr. John Conly (University of Calgary). Mouse adapted SARS-CoV2 was previously described in.^10^ Briefly, a full-length molecular clone “Wuhan variant SARS-CoV-2 with D614G mutation in Spike” was purchased from Telesis Bio (formerly Codex DNA) San Diego, CA) in a BAC plasmid backbone. Then three spike protein amino acid changes (Q493K, Q498Y, P499Y) were introduced to increase binding to mouse ACE2. Virus stocks were generated in Vero CCL81. Sendai virus (SeV) Cantell strain was obtained from Charles River Laboratories (Wilmington, MA). Human coronavirus 229E (VR-740) were obtained from the American Type Culture Collection (Manassas, VA). Virus stocks were generated and titrated (by plaque assay) in the following cell lines: HCoV-229E in Huh7 cells; Mayaro virus and Zika virus in Vero CCL81 cells. SARS-CoV (Tor2 isolate) was provided by Dr. Nathalie Bastien at the National Microbiology Laboratory (Winnipeg, MB) and propagated on Vero E6 cells in the Containment Level 3 Facility at VIDO, University of Saskatchewan (Saskatoon, SK).

#### Viral molecular clones

Viral clones were generated and produced as described in.^38^ The mutant virus spike (SpikeΔ9) was generated by mutating the Fragment #8 using the Q5 Site-Directed Mutagenesis Kit (E0554) from New England Biolabs. Using the following primers: Forward Primer AGAGGTTTGATAACCCTG Reverse Primer TAGCATGGAACCAAGTAAC.

#### SARS-CoV-2 Replicon

The CMV promoter driven SARS-CoV-2 delta variant subgenomic replicon was synthesized by Telesis Bio (formerly Codex DNA). Briefly, the replicon was designed by replacing Spike and envelope protein genes with cassettes for Zeocin resistance and nanoluciferase containing an IL-6 signal sequence for secretion. In addition, a Neon Green fluorescent protein cassette followed by a Thosea asigna virus 2A (T2A) self-cleaving peptide coding sequence was fused in frame with the nucleocapsid gene.

#### SARS-CoV-2 virus infections

Cells were seeded overnight to achieve 70–90% confluence at the time of infection. Monolayers were infected with the indicated variant of SARS-CoV-2 at the indicated MOIs for 1 h at 37°C in DMEM. After 1 h, the inoculum was removed, and cells were washed and replenished with regular cell culture media depending on the cell line. 24–48 h post-infection, supernatants were harvested and stored at −80°C.

#### Viral titrations

Plaque assay. Vero CCL-81 cells were plated in a 24-well plate (1.0 × 10ˆ5 cells per well) and incubated overnight at 37 °C. Virus-containing media were serially diluted (10−1 to 10−6) with DMEM media into 96-well plates. Then 100 μL of each dilution was added in duplicate to Vero cells in the 24-well plates and samples were incubated at 37 °C in 5% CO_2_ for 1 h with rocking every 15 min to prevent cells from drying out. After 1 h incubation, the virus-containing media were removed from the Vero cells in the 24-well plates and 1 mL of pre-warmed plaquing media (MEM media containing 2% FBS and 0.75% carboxymethylcellulose) was added to each well. Plates were incubated at 37 °C in 5% CO_2_ for 3 days to allow plaque formation. On day three, cells were fixed by adding 1 mL of 4% formaldehyde solution in PBS to each well. After incubation at room temperature for 30 min, the fixative was removed, and wells were washed with water and 1 mL of staining solution (1% (w/v) crystal violet in 20% methanol) was added to each well. The staining solution was removed after 30 min and plates were washed with water until the plaques were visible. Plaques were counted from wells containing 5–30 plaques. Titers were calculated in PFU/mL using the following formula: Titer (PFU/mL) = number of plaques counted × 10ˆdilution counted/volume of inoculum (in mL). TCID_50_. SARS-CoV was titrated by TCID_50_ in Vero CCL81 cells as described.^66^

#### Genome-wide CRISPR-Screen

The human CRISPR Brunello genome-scale CRISPR library (Addgene # 52963) was used to perform the pooled CRISPR knockout screen in HEK293T-ACE2-Cas9 cells following the protocol described in.^14^ Briefly, host cells were transduced with lentivirus at an MOT of 0.3. The cells were then selected using Puromycin 1 μg/mL for 6 days. Finally, the selected cells were infected three times with SARS-CoV-2 with an MOI of 3 at 48h intervals and the surviving cells were harvested, and the genomic DNA was extracted using Quick-gDNA MidiPrep (Zymo Research, cat. no. D3100). Genomic DNA was then used to prepare sequencing libraries. Libraries were then sequenced at NOVOGENE Corporation in an Illumina NovaSeq 6,000 Paired End 150 at a sequencing dept of at least 10 million reads per sample. Guide RNA counts were processed using the MAGeCK pipeline with an output of RRA *p*-values and gene ranks.^17^

#### Generation of cell lines

293T-ACE2 NAE1 KO and 293T-ACE2 FGFR1 KO were generated by transduction using lentivirus encoding their respective sgRNA. After transduction, polyclonal populations were prepared by selection using Puromycin. Clonal cell lines were generated by amplifying single cell colonies from the polyclonal populations. 293T-ACE2 TMPRSS2-V5 and 293T-ACE2 FGFR1-V5 Cell lines were generated by transduction using lentivirus encoding their respective ORFs. After transduction, polyclonal populations were prepared by selection with puromycin.

#### Plasmids

NAE1 sgRNA (Cat#90777), FGFR1 sgRNA (Cat#76067) plasmids were obtained from Addgene. Plasmids encoding V5-tagged proteins were obtained from the ORFome library (Human TRC 3.0 arrayed ORF library – CSTORFG) MilliporeSigma Canada Ltd. Split GFP plasmids were obtained from Addgene, sGFP-C is pBiFC-bFosVC155 (Cat#22013) and sGFP-N is pBiFC-bJunVN155(I152L) (Cat#27098). pcDNA5/TO Mammalian Expression Vector was sourced from ThermoFisher. Plasmid expressing codon optimized Spike D614G was also obtained from Addgene (Cat#177960). SpikeΔ9 plasmid was generated by deleting nucleotides 202–229 using the Q5 Site-Directed Mutagenesis Kit (E0554) from New England Biolabs using the following primers: Forward Primer: 5′AAGCGGTTCGACAATCC Reverse Primer: 5′GGCGTGGAACCAGGTC.

#### Syncytia forming assay

We used plasmids encoding codon optimized Spike WT or SpikeΔ9 under CMV promoter. Plasmid DNAs were electroporated into H23-ACE2 cells as per the following protocol. Ten million cells were resuspended in 1 mL of 25 mM HEPES (pH 7.6), 10 mM KH_2_PO_4_, 120 mM KCl, 0.15 mM CaCl_2_, 2 mM ethylene glycol tetra acetic acid (EGTA), 5 mM MgCl_2_, 5 mM adenosine triphosphate (ATP), and 2 mM L-glutamine. 400 μL of cell suspension and 10 μg of plasmid DNA at 100 ng/μL (1:1 M ratio between Spike and Split GFP or pcDNA 5TO plasmids) were used in each electroporation using 4 mm cuvettes (Bio-Rad) at 330 V, 975 μF. H23-ACE2 cells were electroporated with either (sGFP-N + Spike), (sGFP-C + Spike), (sGFP-N + Spike Δ9), (sGFP-C + Spike Δ9), (sGFP-N + 5TO) or (sGFP-C + 5TO). The following combination of cells were mixed in a 1:1 ratio and seeded in 24 well plates. a. (sGFP-N + Spike) and (sGFP-C + Spike), b. (sGFP-N + Spike Δ9) and (sGFP-C + Spike Δ9), c. (sGFP-N + 5TO) and (sGFP-C + 5TO). The drug treatments were done as described in the figure legends 4h post electroporation. Finally, the cells were imaged 24h post-treatment using an EVOS M7000 (ThermoFisher) microscope, using the 4X objective, 55 images were acquired per well and stitched together to cover the full area of the well. The images were acquired in two channels, brightfield and GFP. Analysis was performed calculating total intensity in ImageJ software, values were further processed using GraphPad Prism software.

#### Lentivirus production

All lentiviruses were produced in 293FT cells using PEI for transfection. Following the protocol as described here.^14^

#### Structural modeling

The structural models for SARS-CoV2 S-protein NTD from the wild type (NTD-WT), and the mutant NTDΔ68–76 was built using ColabFold.^67^ ColabFold v1.5.5, an optimized implementation of AlphaFold2 (AF2), including AlphaFold Multimer for predicting protein complexes, was executed within a Google Colab notebook.^68^^,^^69^ ColabFold accelerates protein structure and complex predictions by combining the fast homology search of MMseqs2 with AlphaFold. The Colab notebook, a cloud-based platform for coding and running programs, leveraging Amber99sb force fields,^70^ which are parameters necessary to ensure accurate peptide bond geometry accuracy after prediction. Each run used specific settings such as the multimer_V2 weights, 48 recycles and yielded 5 models for analysis. AlphaFold assessed the intra-domain confidence or the quality of protein as a per-residue predicted local distance difference test (pLDDT) score. The predicted template modeling score (pTM) represents the global confidence score for the entire model, rather than just individual domains, and depends on the quality of the reference structures (templates) used for modeling. The pTM score >0.5 is considered high enough to make a reliable inference. The pLDDT scores and pTM for NTD-WT (pLDDT = 87.4 pTM = 0.867) and NTDΔ68–76 (pLDDT = 87.1 pTM = 0.864). The structures were visualized and analyzed using PyMOL v.2.5.4 (Schrödinger, LLC.).^71^ For modeling the S1 trimer in the closed conformation, AlphaFold 3^72^ was used, as the residue length (2055 residues) prevented ColabFold from running effectively.

#### Isolation of MLN4924-resistant mutants

Vero CCL81 cells were seeded in 6 wells plates and treated with either DMSO or MLN4924. Twenty-four hours post treatment, cells were infected with SARS-CoV-2 at an MOI of 0.01 for either 72h or an observed 40% CPE. At that point the supernatant was harvested and used for subsequent rounds of infections and titrations. SARS-CoV-2 resistant to MLN4924 was used to infect Vero CCL81 and total RNA was extracted 24h post-infection. The SARS-CoV-2 genomes were sequenced by NGS techniques using the QiaSeq Direct SARS-CoV-2 library preparation kit (Qiagen, ID: 333891), followed by sequencing at NOVOGENE Corporation in an Illumina NovaSeq 6,000 Paired End 150 at a sequencing dept of at least 10 million reads per sample. Full length genomes were assembled from raw reads using the CLC Genomics Workbench 20 software.

#### Assessing SARS-CoV-2 replication activity

The SARS-CoV-2 Delta NLuc reporter replicon plasmid was transfected into 293T-ACE2 cells using Lipofectamine 2000 following manufacturer’s recommendations. Twenty-four-hour post transfection, cell media was replaced with fresh media with the designated inhibitors. To assess the replication activity, 100 μL of supernatant were collected to measure Nano Luciferase luminescence at the stated time points using the Promega Kit Nano-Glo Luciferase Assay System. Luminescent was recorded at an integration of 1 s per well using Synergy HTX plate reader (Agilent).

#### Cell viability

Cells were grown in clear bottom, opaque walls 96 wells plates (Greiner Bio One, ref. 655083). Forty-eight hours post drug treatment, cell viability was measured using a CellTiter-Glo Luminescent Cell Viability Assay kit (Promega, REF G7571) following manufacturers recommendations. Luminescent was recorded at an integration of 1 s per well using Synergy HTX plate reader (Agilent). Cell viabilities were assessed under the same treatment conditions in all relevant cell types. For all cell lines used in this manuscript only drug concentrations that retained >90% cell viability were used for the antiviral assays.

#### ELISA

NHBE cells were grown in 96 well plates. 24 h later were treated with DMSO or BGJ398 and 24 h after that infected with Sendai virus (400HU) for 4 and 8 h. ELISA procedure followed the manufacturer instructions (Cat# DY008B). Optical density was recorded at 450 and 540 nm using Synergy HTX plate reader (Agilent).

#### Illustrations

Figures 1A, 2B, 3A, and 5A were created using the Biorender software (Biorender.com). ScatterPlot in Figure 1B was created with R-Studio using ggplot2. Figure S8 was created with SnapGene software.

#### *In Vivo* animal experiments

Animal experiments were approved by the University of Alberta Animal Care and Use Committee (AUP00003963). All SARS-CoV-2 infection studies were conducted in a certified BSL3 containment facility at the University of Alberta. Briefly, 6–8 weeks old female BALB/c mice (Charles River laboratories) were treated with appropriate drug dosage (determined by pilot maximum tolerable dose experiment) intranasally on days −2 and −1 pre-challenge in a total volume of 50 μL (25 μL per nare). On day 0, mice were inoculated intranasally with mouse adapted strain of SARS-CoV-2 at a dose of 3.0 × 10^3^ PFU in a total volume of 50 μL (25 μL per nare). On days +1 and +2 post-challenge, mice were intranasally treated again with the same drugs. Tissue samples were collected on day 4 post-challenge for virus titration and histopathology. Mice were monitored daily for signs of infection and morbidity for a period of 14 days. Animals who lost greater than 20% of their initial body weight were to be humanely euthanized and excluded from the study; however, no animals exceeded this threshold.

#### Antibodies and compounds

V5 Tag Monoclonal Antibody (Cat# R960-25) was bought from Thermofisher. Β-Tubulin antibody was bought from Cell signaling (Cat# 2146), Anti-ACE2 antibody [EPR4435(2)] was bought from abcam. Goat anti-rabbit conjugated to Alexa 680 (A21076), Donkey anti-rabbit conjugated to Dylight 800 (SA5-10044) Goat anti-mouse conjugated to Alexa 750 (A-21057) were purchased from Life Technologies. Dimethyl sulfoxide (DMSO) was purchased from Sigma-Aldrich, IFNα was purchased from abcam (ab48750).

#### Inhibitors

BGJ398 (Catalog#S2183), MLN4924 (Catalog#S7109), Tannic Acid (Catalog#S3951), Binimetinib (Catalog#S7007), TAS4464 (Catalog#S8849), PD98059 (Catalog#S1177), SCH772984 (Catalog#S7101), HS-173 (Catalog#S7356), SP600125 (Catalog#S1460), DC661 (Catalog#S8808), GSK923295 (Catalog#S7090), Aprotinin (Catalog#S7377), U73122 (Catalog#S8011), Remdesivir (Catalog# S8932) were purchased from Sellekchem. Cpd7a (Catalog#508957), NFAT inhibitor (Catalog#N7032), OGG1 inhibitor O8 (Catalog#SML1697) was from Milipore Sigma. SGC-AAK1-1 (Catalog#6528) was from TOCRIS. Compounds were dissolved in DMSO or Milli-Q Water to produce working stocks which were stored at −80 °C until used in experiments.

#### Immunoblotting

At designated points post-infection or post-transfection, cells were washed twice with phosphate-buffered saline (PBS) before lysing with 2X SDS sample buffer with 100 mM dithiothreitol (DTT). The samples were incubated at 98^0^C for 10 min to denature proteins which were then separated by SDS-PAGE and transferred to polyvinylidene difluoride membranes for immunoblotting. The membranes were incubated with blocking solution (5% bovine serum albumin [BSA; Sigma Aldrich] in PBS-0.05% Tween 20) for 30 min before exposure to primary antibodies diluted in Blocking solution for 60 min. Following three washes with PBS-0.05% Tween 20 for 10 min each, the blots were incubated with secondary antibodies in Blocking solution for 60 min. The blots were washed three times with PBS-0.05% Tween 20, once with PBS and then imaged with an Odyssey Infrared Imaging system. Quantification of proteins was performed using Odyssey Image Studio Lite Software Version 5.2.

#### Quantitative real-time PCR (qRT-PCR)

Total RNA was isolated from cells using the RNA NucleoSpin Kit (Machery Nagel) and reverse transcribed using random primers (Invitrogen) and Improm-II reverse transcriptase (Promega) at 42°C for 1.5 h to generate cDNAs. The cDNAs were diluted 1:5 with water and 5% volume was mixed with the appropriate primers (Integrated DNA Technologies) and the PerfecTa SYBR green SuperMix with Low ROX (Quanta Biosciences) and amplified for 40 cycles (30 s at 94°C, 40 s at 55°C and 20 s at 68°C) in a Biorad CFX96 qRT-PCR machine. The gene targets and primer sequence are listed in Table 2. CT values were normalized using *ACTB* mRNA as the internal control. The ΔΔCT values were determined using control samples as the reference value. Relative levels of mRNAs were calculated using the formulas 2^(−ΔΔCT)^.

### Quantification and statistical analysis

#### Statistical analysis

All statistical analyses were performed using GraphPad Prism software. Paired Student’s *t* test was performed for pairwise statistical comparison while one-way ANOVA was used for comparison of multiple samples. The mean ± standard error of the mean is shown in all bar and line graphs.

## Supplemental information

Document S1. Figures S1–S16

Document S2. Immunoblots raw files

Document S3. Microscopy raw files
