# Supplementary material for: FGFR signaling and neddylation facilitate SARS-CoV-2 infection by modulating interferon induction and viral entry, respectively
Source: iScience. 2025 Dec 29;29(2):114566. doi: 10.1016/j.isci.2025.114566 (PMC12828524; doi:10.1016/j.isci.2025.114566)
Supplement: Document S1. Figures S1–S16 [file mmc1.pdf]

iScience, Volume *29*

Supplemental information

FGFR signaling and neddylation facilitate SARS-CoV-2 infection by modulating interferon induction and viral entry, respectively

Alberto Felix-Lopez, Joaquin Lopez-Orozco, Mohamed Elaish, Nawell Fayad, Zaikun Xu, Tekeleselassie Woldemariam, Bardes B. Hassan, Rashmi Panigrahi, Juveriya Qamar Khan, Megha Rohamare, Irv Mayers, J.N. Mark Glover, Joyce A. Wilson, Darryl Falzarano, Anil Kumar, and Tom C. Hobman

SUPPLEMENTAL FIGURES

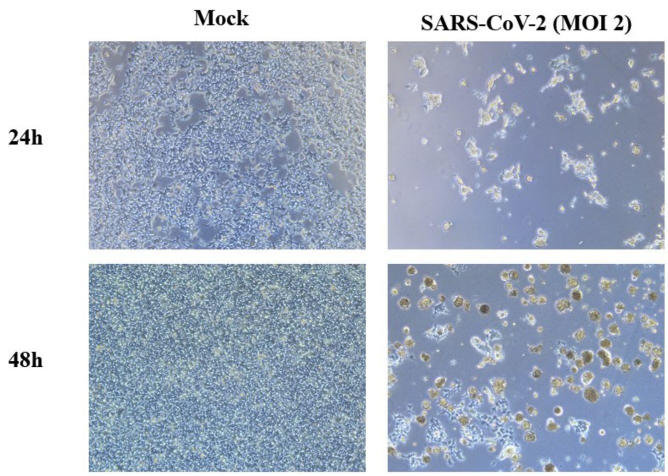

Figure S1. Cytopathic effects induced by SARS-CoV-2 infection in HEK293T-ACE2 cells.

Related to figure 1

Representative microscopic images of HEK293T-ACE2 cells infected with SARS-CoV-2 (72B/CA/CALG) at an MOI of 2. Images were captured 24- and 48-hours post-infection to illustrate the cytopathic effect (CPE) in the cells.

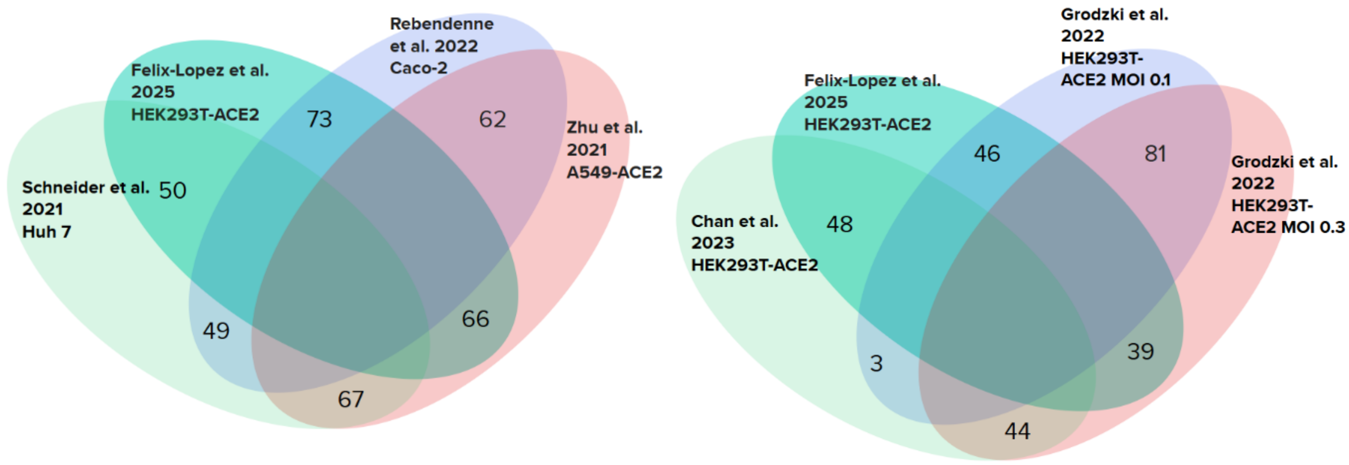

Figure S2. Comparing data from different CRISPR-KO screens for host-dependency factors for SARS-CoV-2.

Related to Figure 1

Comparison of top 1000 hits from different CRISPR KO screens. Venn diagram showing the overlap between host dependency factors for SARS-CoV-2 identified in CRISPR KO screens using the Brunello library (left) or HEK293T-ACE2 cells (right). The overlapping region represents host dependency identified in both screens.

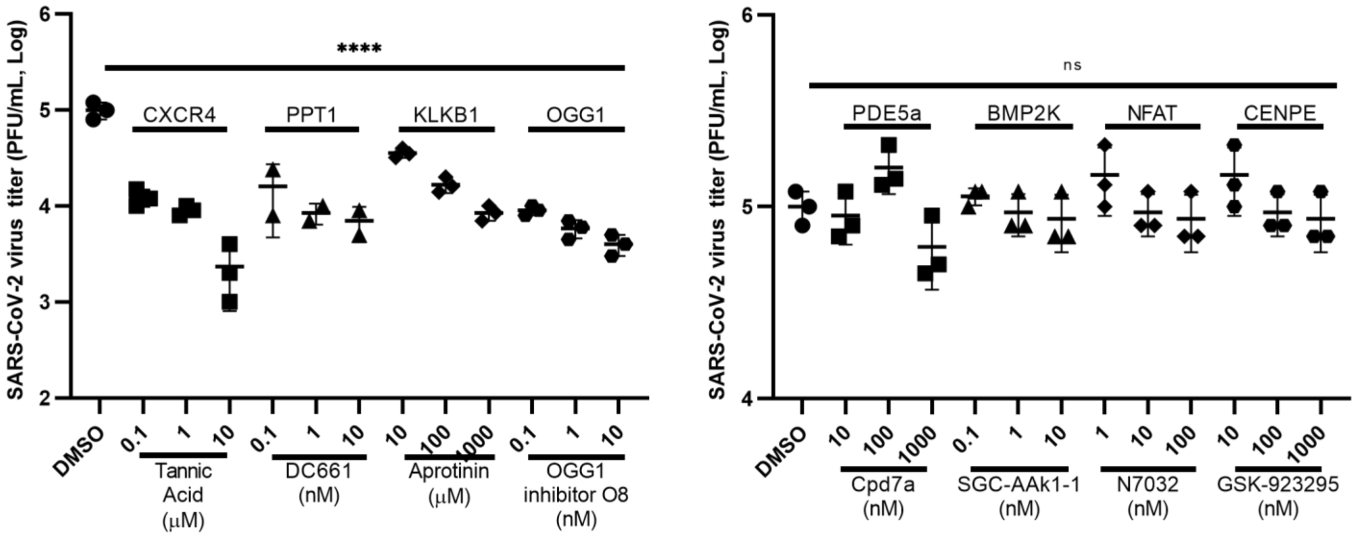

Figure S3. Effect of inhibiting other top 10 (from CRISPR screen) host cell factors on SARS-CoV-2 replication.

Related to Figure 1

HEK293T-ACE2 cells were pre-treated with the inhibitors that target indicated host factors for 24 h and then infected with SARS-CoV-2 (72B/CA/CALG) using MOI of 0.1. Twenty-four hours later, media were collected and subjected to plaque assay to determine viral titers. Data shown is averaged from three independent experiments. Error bars represent the standard error of the mean. One-way ANOVA with Dunnett's multiple comparison test was used to determine statistical significance between control (DMSO) and drug-treated samples. P value <0.0001 ****, ns = not significant.

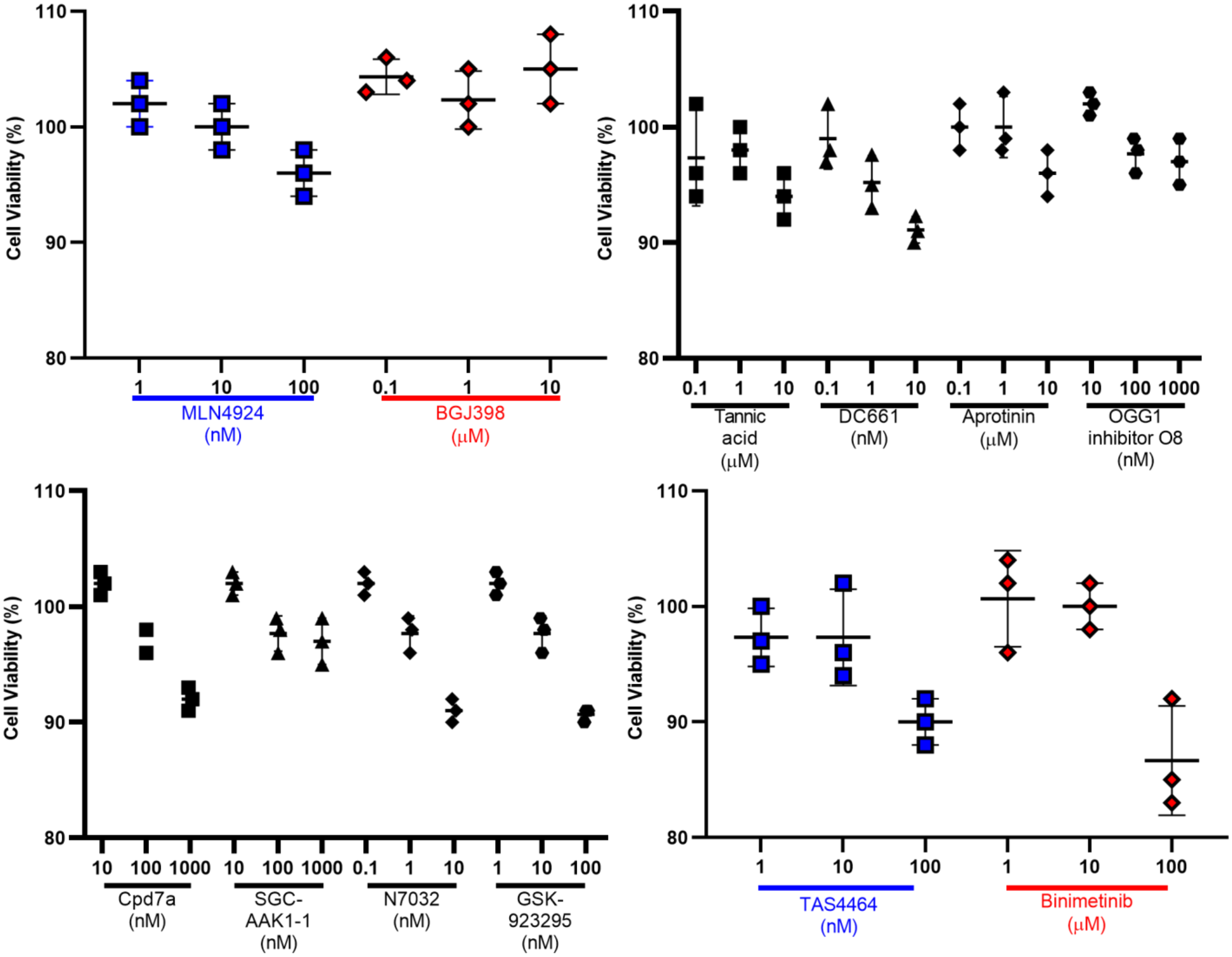

Figure S4. Effect of host dependency factor inhibitors on HEK293T-ACE2 viability.

Related to Figure 1

Cell viability of HEK293T-ACE2 cells treated host cell inhibitors for 48 h at indicated concentrations compared to vehicle (DMSO) treated cells. Error bars represent standard error of the mean.

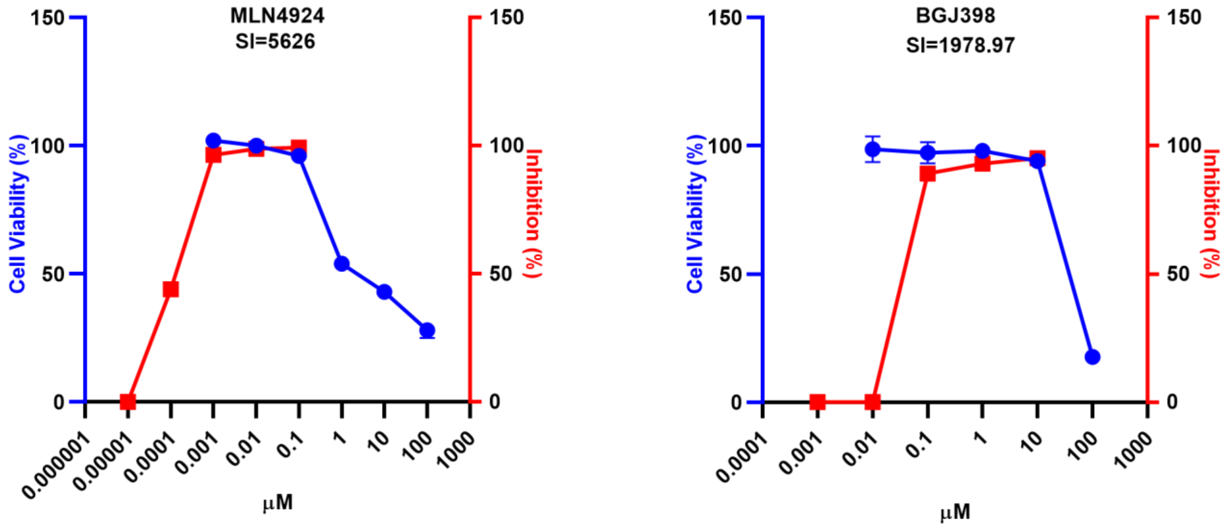

Figure S5. Selectivity Indexes of MLN4924 and BGJ398 in HEK293T-ACE2 cells infected with SARS-CoV-2.

Related to Figure 1

Selectivity indexes (SI) were calculated using the CC50 and IC50 values of MLN4924 or BGJ398 against SARS-CoV-2 (72B/CA/CALG) infection (MOI of 0.1) in HEK293T-ACE2 for 24 hours.

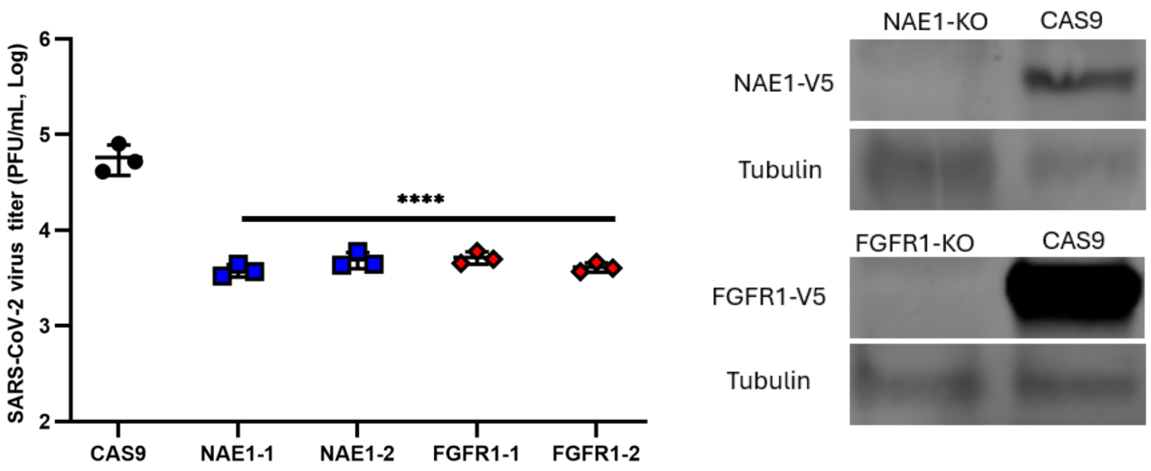

Figure S6. SARS-CoV-2 replication is reduced in HEK293T-ACE2 cells lacking expression of NAE1 or FGFR1.

Related to Figure 1

Left. Two clonal NAE1 KO and FGFR1 KO cell lines generated in HEK293T-ACE2 and their parental HEK293T-ACE2 Cas9 cells were infected with SARS-CoV-2 (72B/CA/CALG) using MOI of 0.1. Twenty-four hours later, media were collected and subjected to plaque assay to determine viral titers. Data shown is averaged from three independent experiments. Error bars represent standard error of the mean. One-way ANOVA with Dunnett's multiple comparison test was used to determine significance between samples treated with cells expressing CAS9 only and those transfected with sgRNAs that inactivate putative host dependency factors for SARS-CoV-2. P value <0.0001 ****.

Right. Immunoblotting was used to assess knockout of NAE1 and FGFR1. Upper section shows HEK293T-ACE2 NAE1 KO or HEK293T-ACE2 Cas9 were transduced with lentivirus expressing NAE1-V5. Lower panel HEK293T-ACE2 FGFR1 KO or HEK293T-ACE2 Cas9 were transduced

with lentivirus expressing FGFR1-V5. V5 antibody was used to detect transduced protein and tubulin antibody as a control.

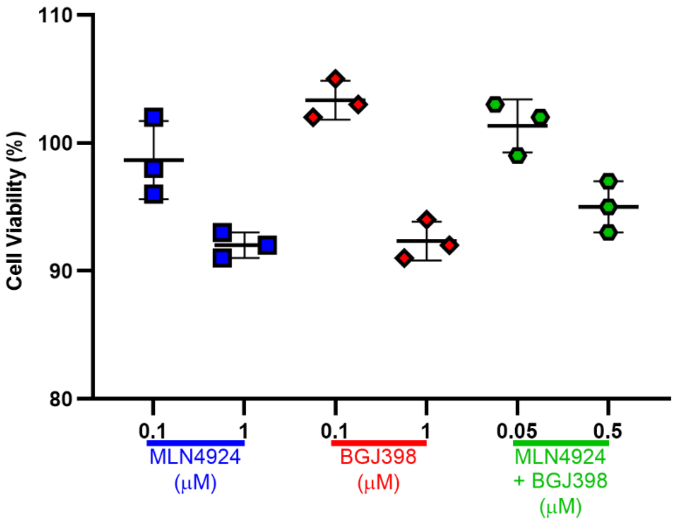

Figure S7. Effect of MLN4924 and BGJ398 on viability of normal human bronchial epithelial cells.

Related to Figure 1

Cell viability of NHBE cells treated with NAE1 and/or FGFR inhibitors for 48 h. Error bars represent standard error of the mean.

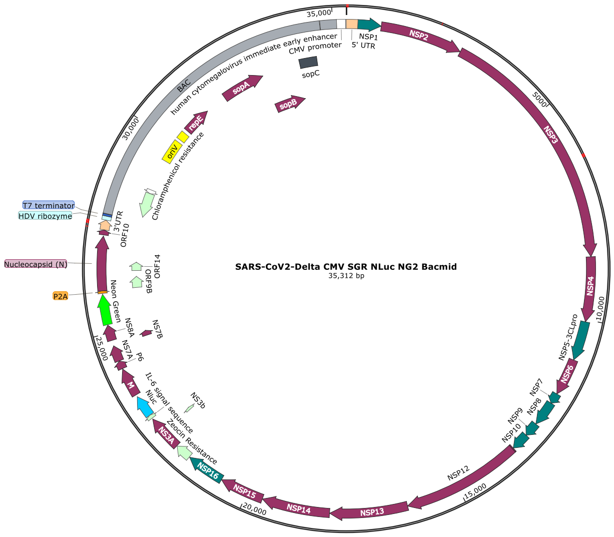

Figure S8. Map of SARS-CoV-2 replicon.

Related to Figure 2

The replicon was designed by replacing Spike and envelope protein genes with cassettes for Zeocin resistance and nanoluciferase containing an IL-6 signal sequence for secretion. In addition, a Neon Green fluorescent protein cassette followed by a Thosea asigna virus 2A (T2A) self-cleaving peptide coding sequence was fused in frame with the nucleocapsid gene.

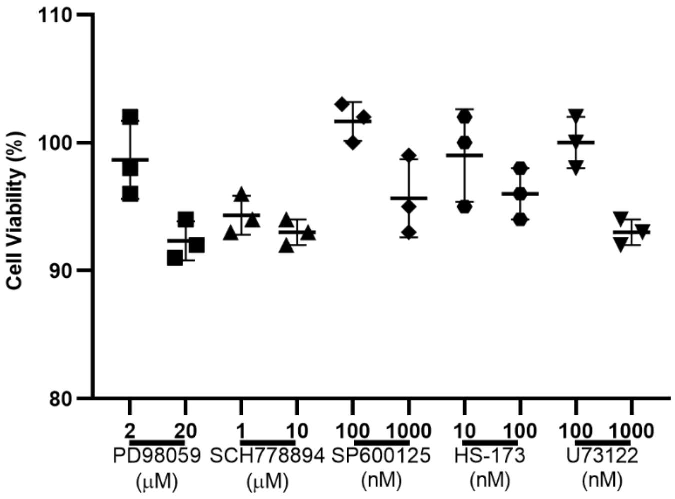

Figure S9. Effect of inhibiting signaling pathways downstream of FGFR1 in HEK293T-ACE2 cells.

Related to Figure 2

Relative cell viabilities of HEK293T-ACE2 were assessed treated for 48 h after treatment with inhibitors of signaling pathway components downstream of FGFR1. DMSO was used as the negative control.

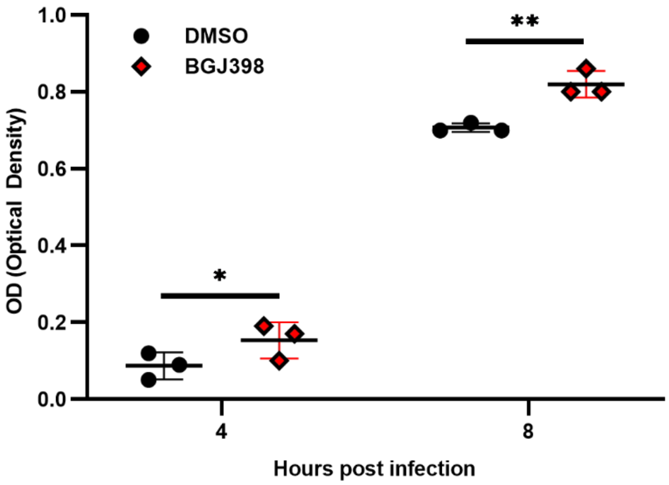

Figure S10. Effect of BGJ398 on IFN β secretion from normal human bronchial epithelial cells.

Related to Figure 2

IFN β secretion was measured using ELISA. Normal human bronchial epithelial cells pre-treated with BGJ398 at 1μM or DMSO for 24h were infected with Sendai virus (400HU) for 4 and 8 hours after which media were collected for ELISA. Paired Student's t-test was used to determine statistical significance between control (DMSO) and BGJ398-treated samples. Error bars represent standard error of the mean. P value *>0.05, **>0.01.

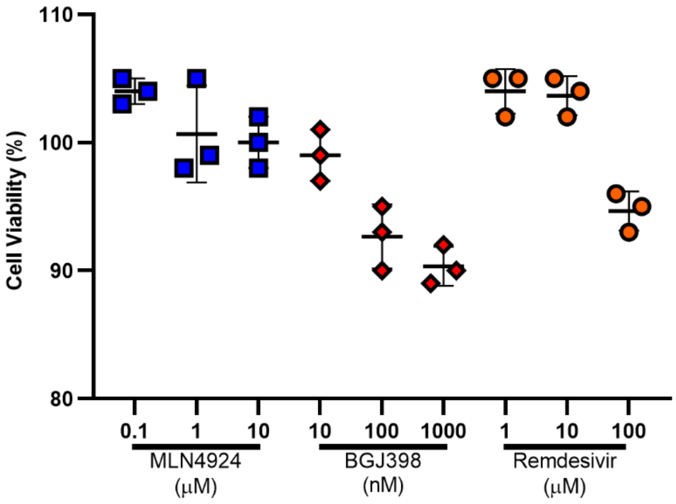

Figure S11. Effect of MLN4924, BGJ398 and Remdesivir on Vero cell viability of Vero.

Related to Figure 2

Vero CCL81 cells treated with the indicated inhibitors and concentrations for 48 hours and cell viabilities relative to DMSO treatment are shown.

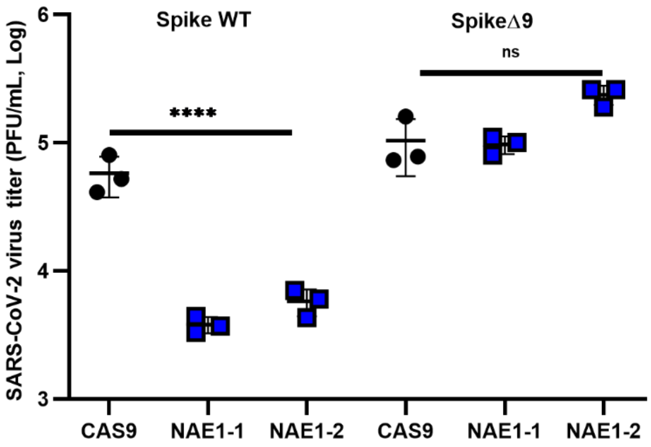

Figure S12. Replication of SARS-CoV-2 SpikeΔ9 is not affected by loss of NAE1.

Related to Figure 3

Two clonal HEK293T-ACE2 NAE1 KO and their parental control HEK293T-ACE2 Cas9 cell lines were infected with SARS-CoV-2 Spike WT or SpikeΔ9 (MOI of 0.1) for 24 hours after which media were collected and subjected to plaque assays to determine viral titers. Error bars represent the standard error of the mean. One-way ANOVA with Dunnett's multiple comparison test was used to determine statistical significance between control (DMSO) and drug-treated samples. P value <0.0001 ****, ns = not significant.

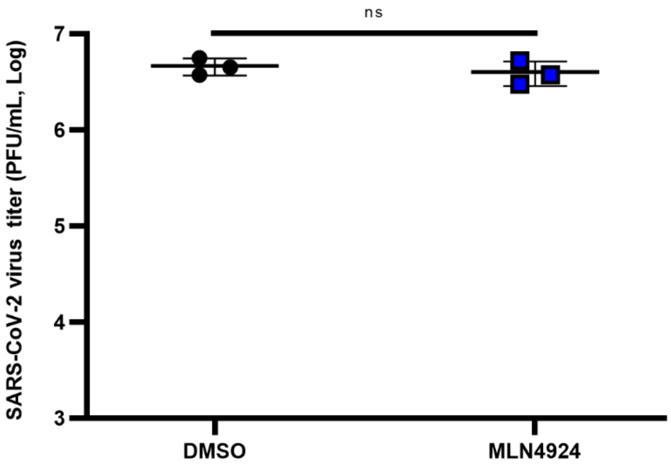

Figure S13. Treatment of cells with MLN4924 after SARS-CoV-2 infection does not viral load.

Related to Figure 4

HEK293T-ACE2 were infected for one hour with SARS-CoV-2 (MOI of 0.1) and then treated with DMSO or MLN4924 (100 nM) 24 hours after which media were collected and subjected to plaque assays to determine viral titers.

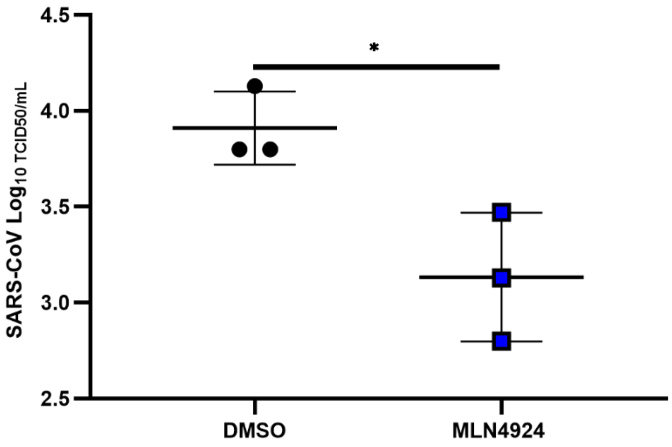

Figure S14. MLN4924 reduces infection of SARS-CoV in Calu-3 cells.

Related to Figure 4

Calu-3 were treated with DMSO or MLN4924 (10 nM) for 24 hours and then infected for one hour with SARS-CoV (MOI 0.1). Twenty-four hours later, media were collected, and viral titers were determined using a $TCID_{50}$ assay. Error bars represent standard error of the mean. Paired Student's t-test was used to determine statistical significance between control (DMSO) and drug-treated samples. Paired Student's t-test was used to determine statistical significance between control (DMSO) and MLN4924-treated samples. P value <0.05 *, ns = not significant.

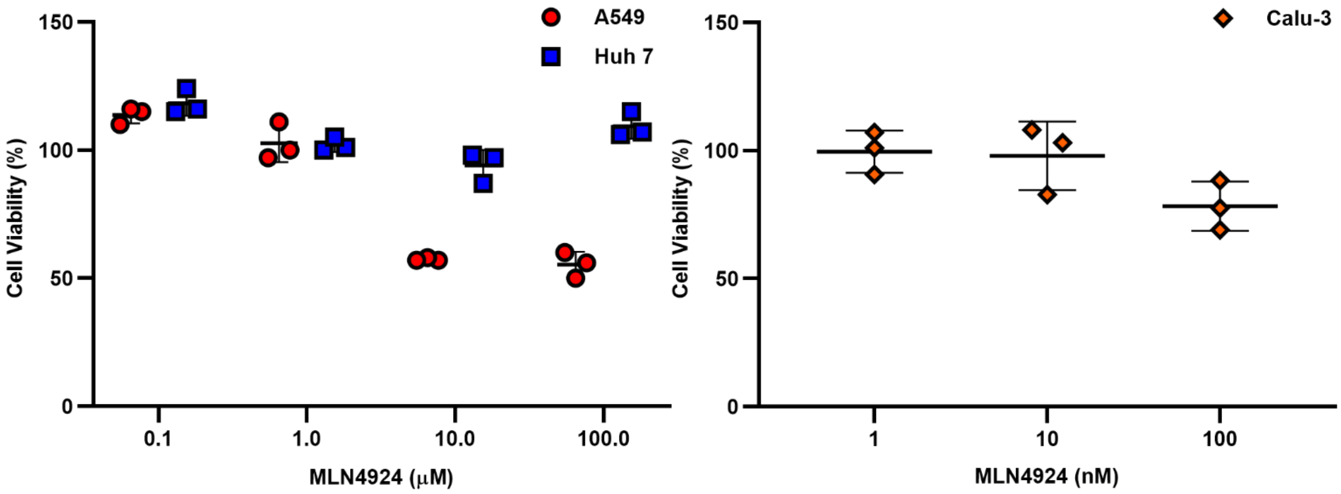

Figure S15. Effect of MLN4924 on viability of A549, Huh 7 and Calu-3 cells.

Related to Figure 4

(Left) A549, Huh 7 and (Right) Calu-3 cells treated with MLN4924 at the indicated concentrations for 48 hours after which cell viabilities were determined relative to DMSO treatment alone. Error bars represent standard error of the mean.

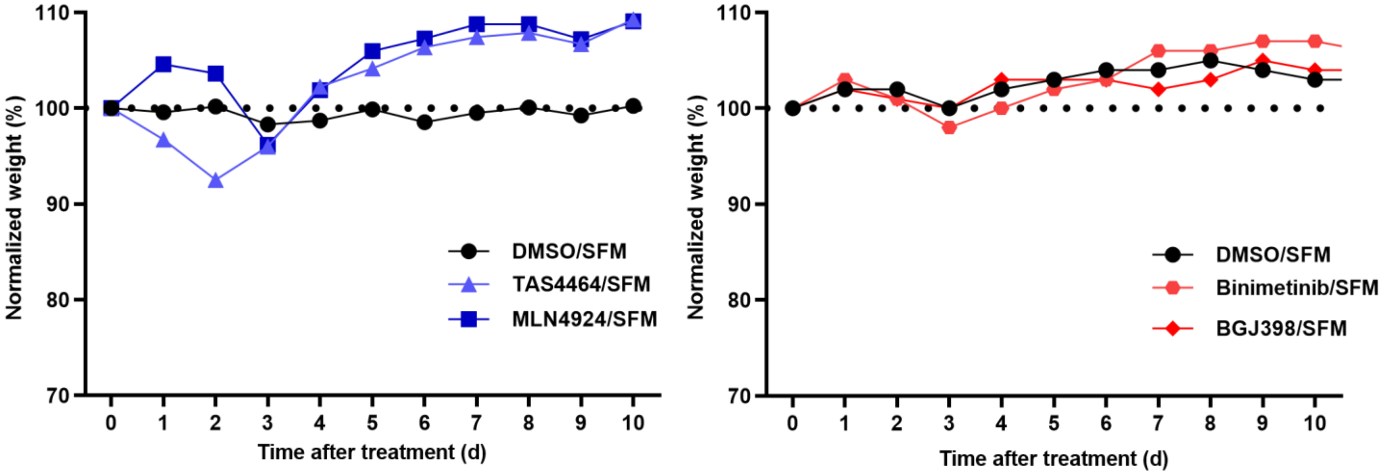

Figure S16. Normalized weight of mice treated with NAE1 or FGFR signaling inhibitors.

Related to Figure 5

Inhibitors of the neddylation pathway (MLN4924, TAS4464) or FGFR signaling (BGJ398, Binimetinib) were administered intranasally to female Balb/c mice (3 mice per group) daily for four days on -2, -1, +1 and +2. After which the body weights of the animals were monitored for 12 days after the first dose. The weights were normalized to mice treated with SFM/DMSO only.
