# Supplementary figures and images for: FGFR signaling and neddylation facilitate SARS-CoV-2 infection by modulating interferon induction and viral entry, respectively
Source: iScience. 2025 Dec 29;29(2):114566. doi: 10.1016/j.isci.2025.114566 (PMC12828524; doi:10.1016/j.isci.2025.114566)

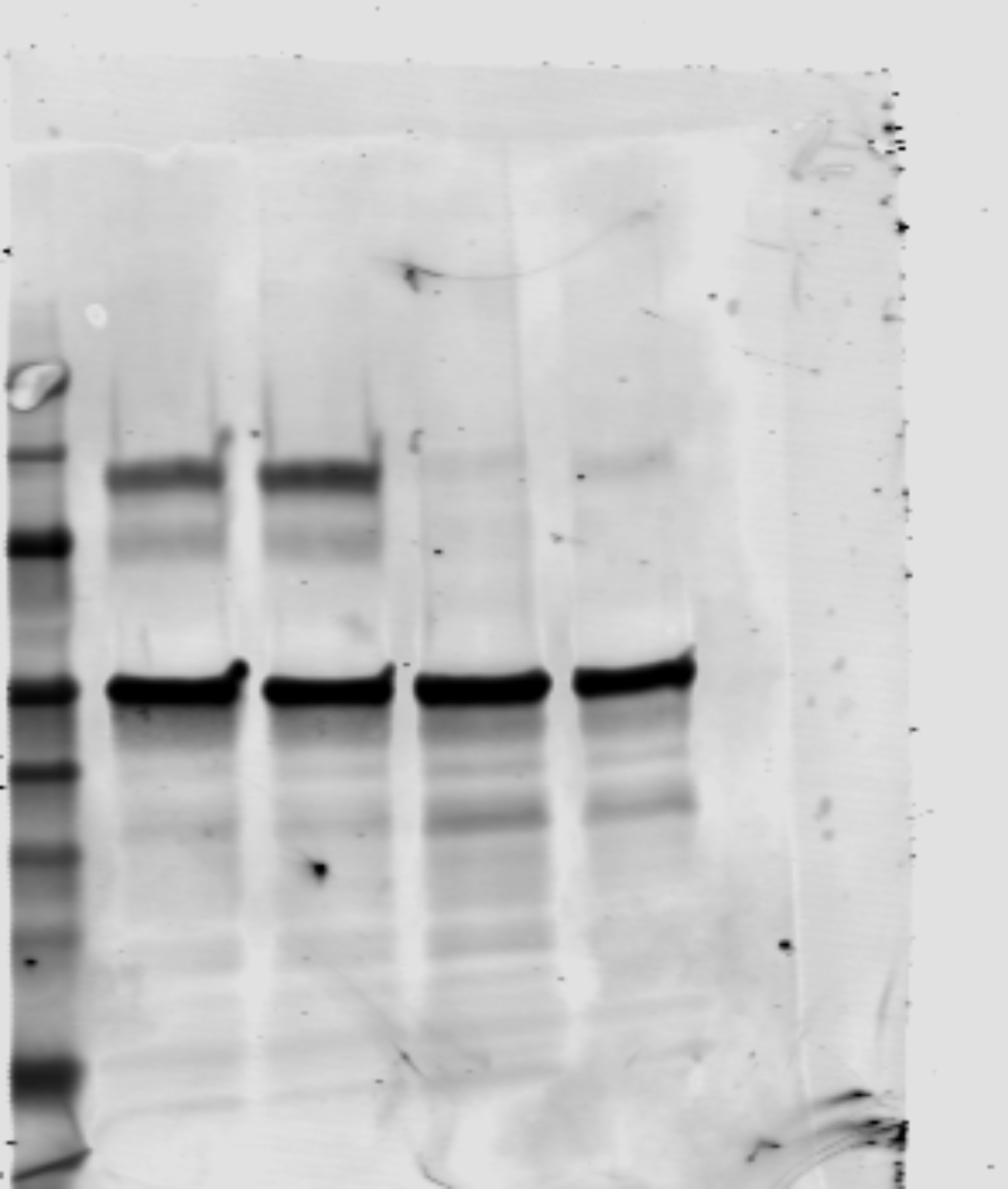

Supplement: Document S2. Immunoblots raw files [file mmc2.zip › WBs/ACE2-Tubulin.tif]

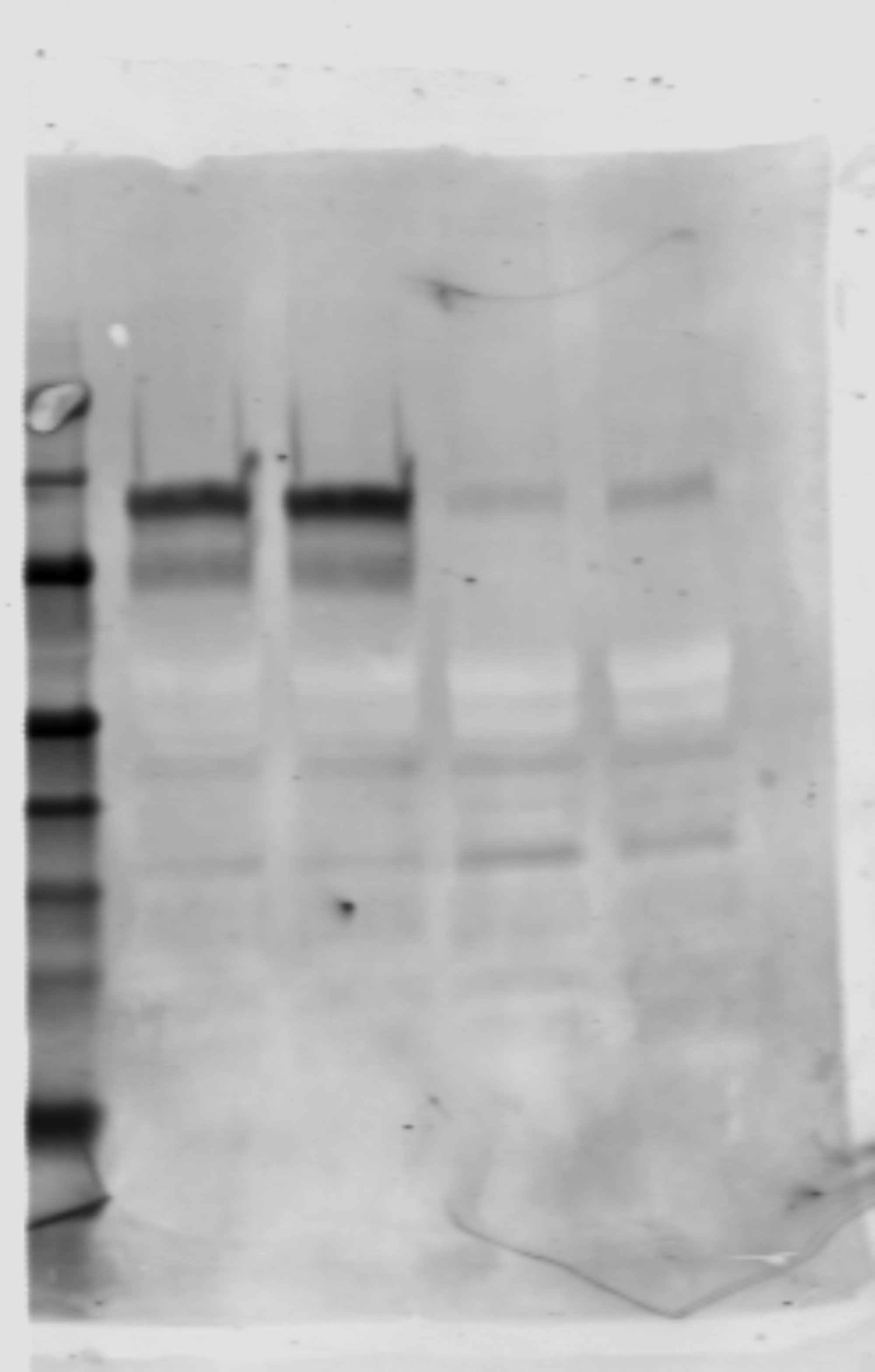

Supplement: Document S2. Immunoblots raw files [file mmc2.zip › WBs/ACE2.tif]

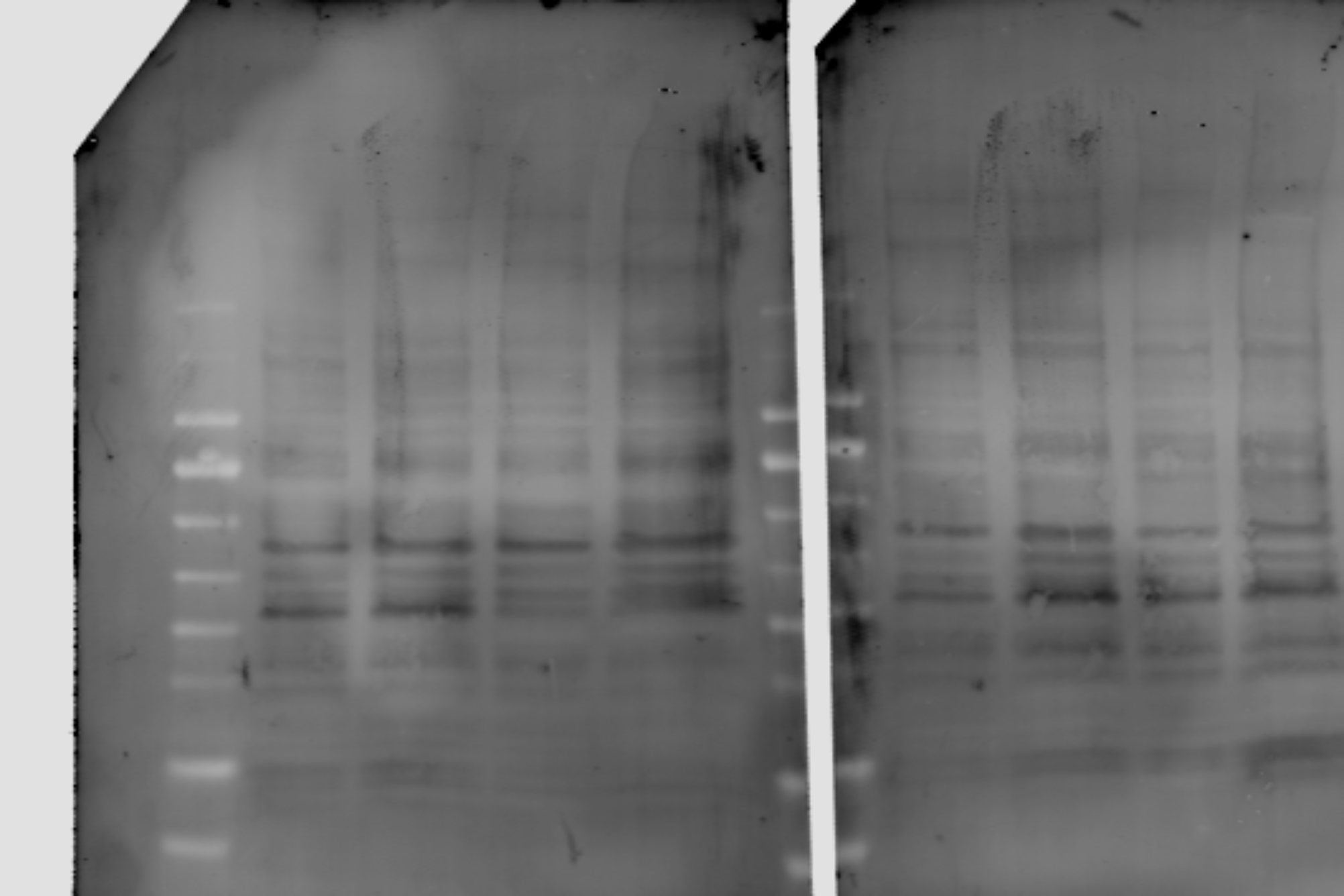

Supplement: Document S2. Immunoblots raw files [file mmc2.zip › WBs/FGFR-KO and NAE1-KO - Tubulin.png]

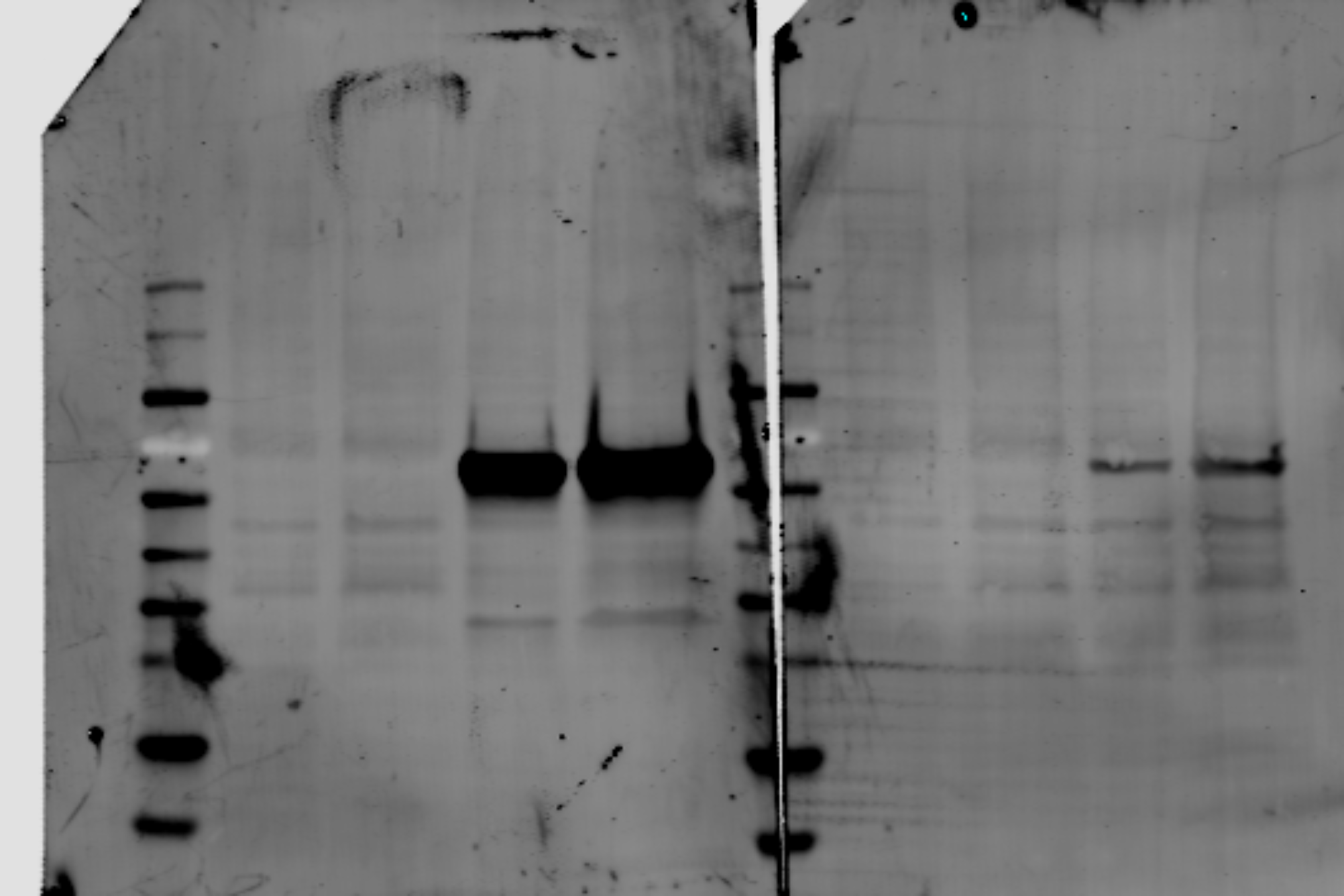

Supplement: Document S2. Immunoblots raw files [file mmc2.zip › WBs/FGFR-KO and NAE1-KO.tif]

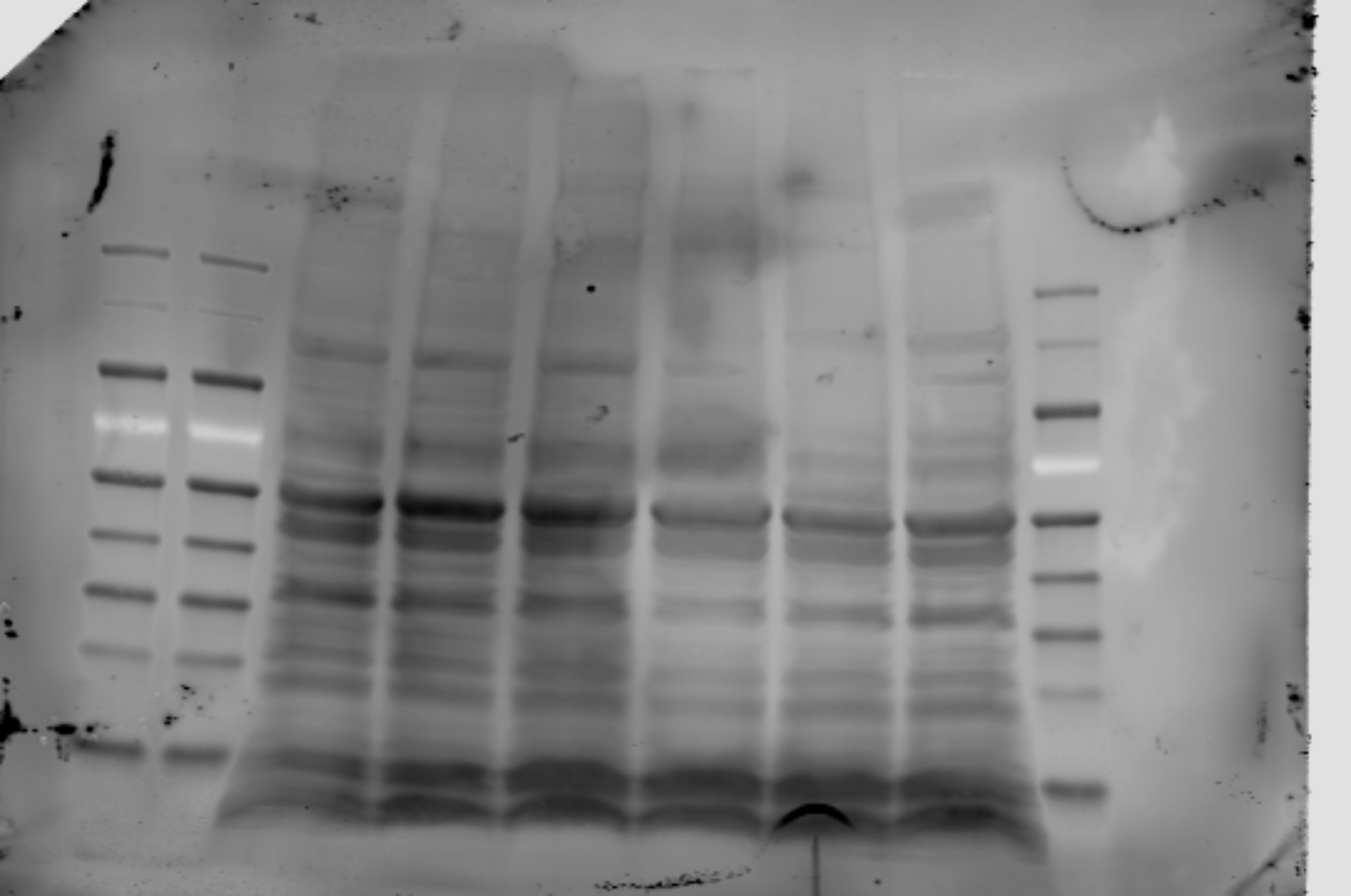

Supplement: Document S2. Immunoblots raw files [file mmc2.zip › WBs/TMPRSS2-tubulin.tif]

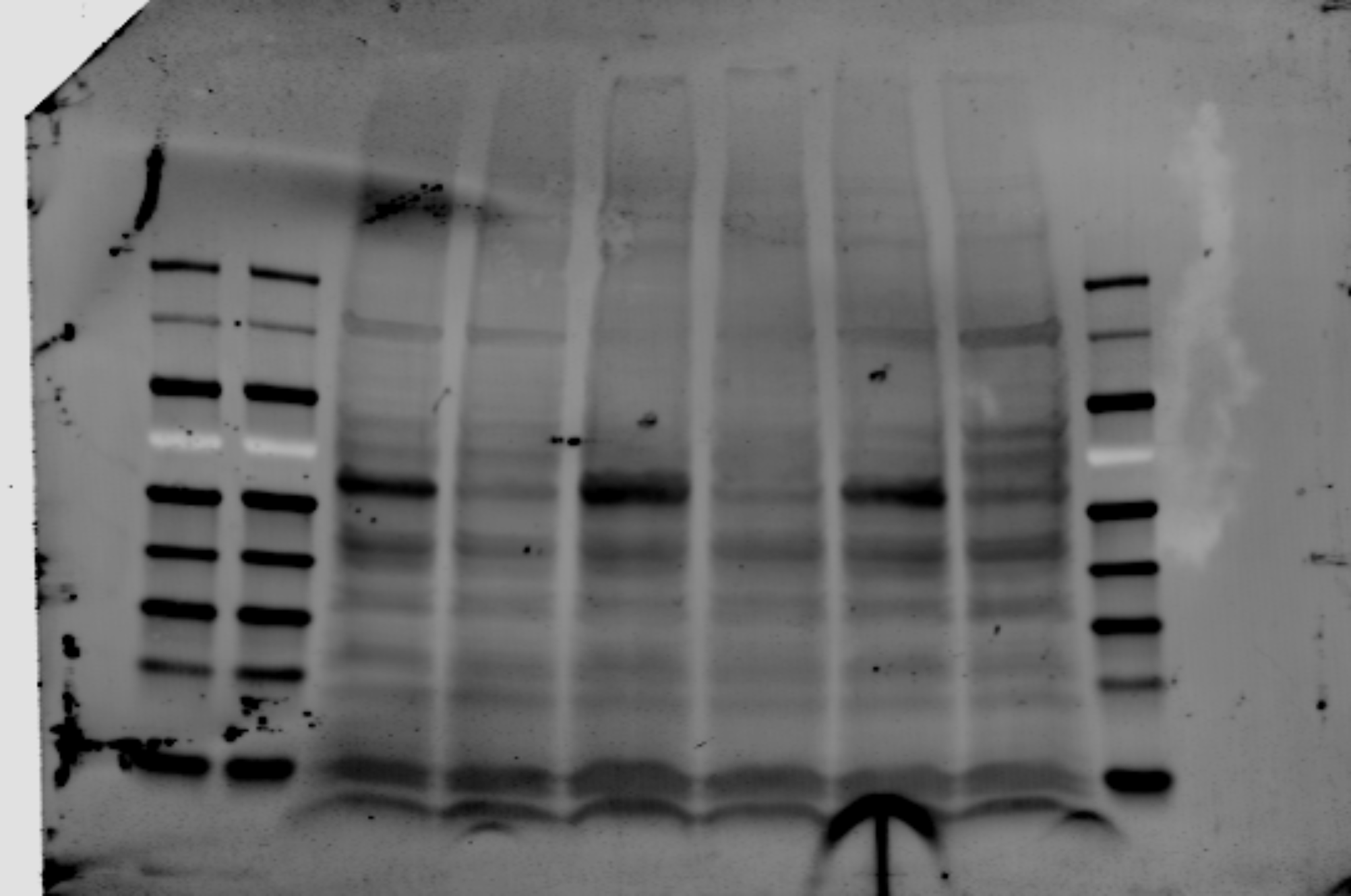

Supplement: Document S2. Immunoblots raw files [file mmc2.zip › WBs/TMPRSS2.png]

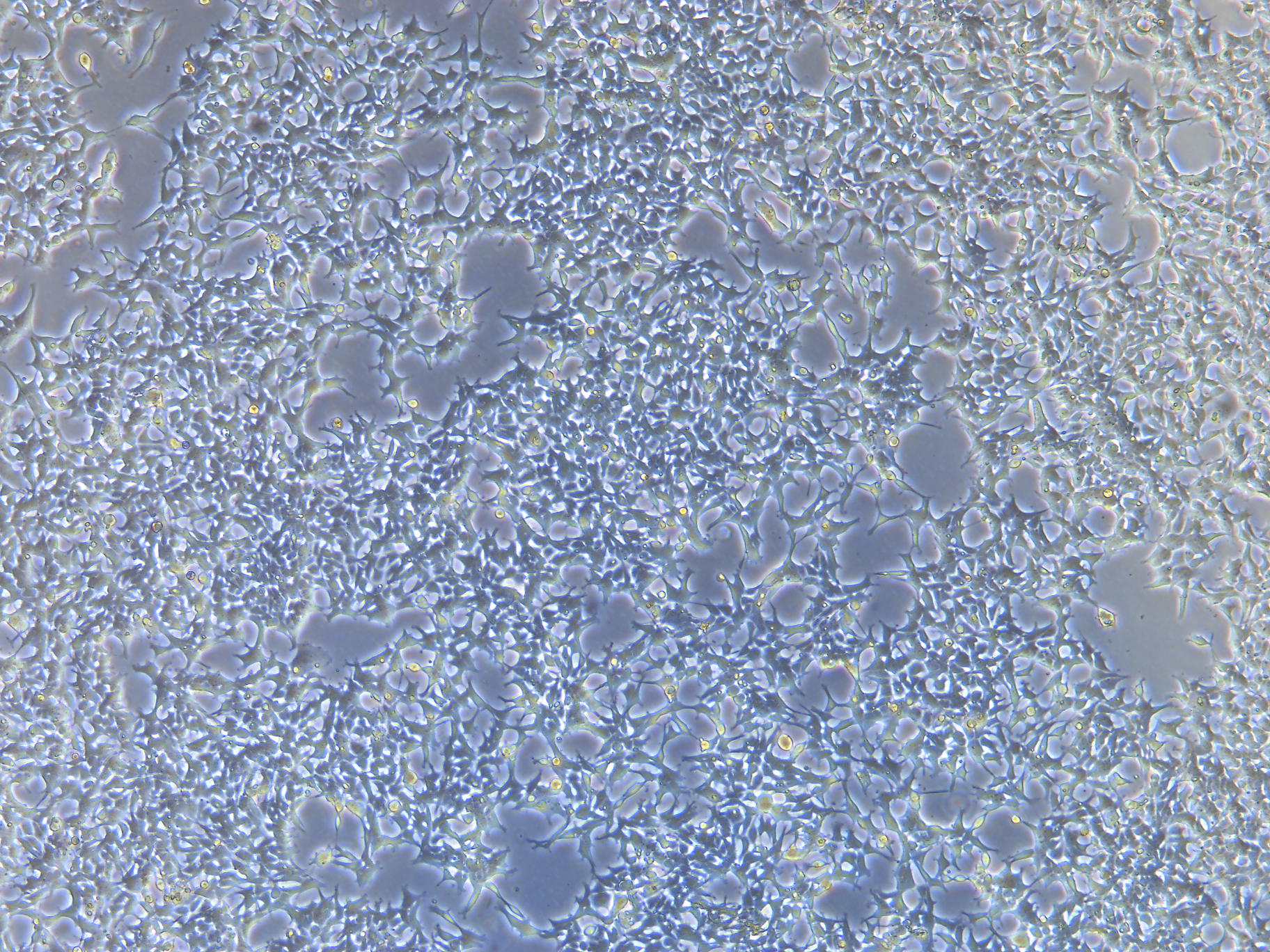

Supplement: Document S3. Microscopy raw files [file mmc3.zip › Microscopy/HEK293T-ACE2 Mock 24h.JPG]

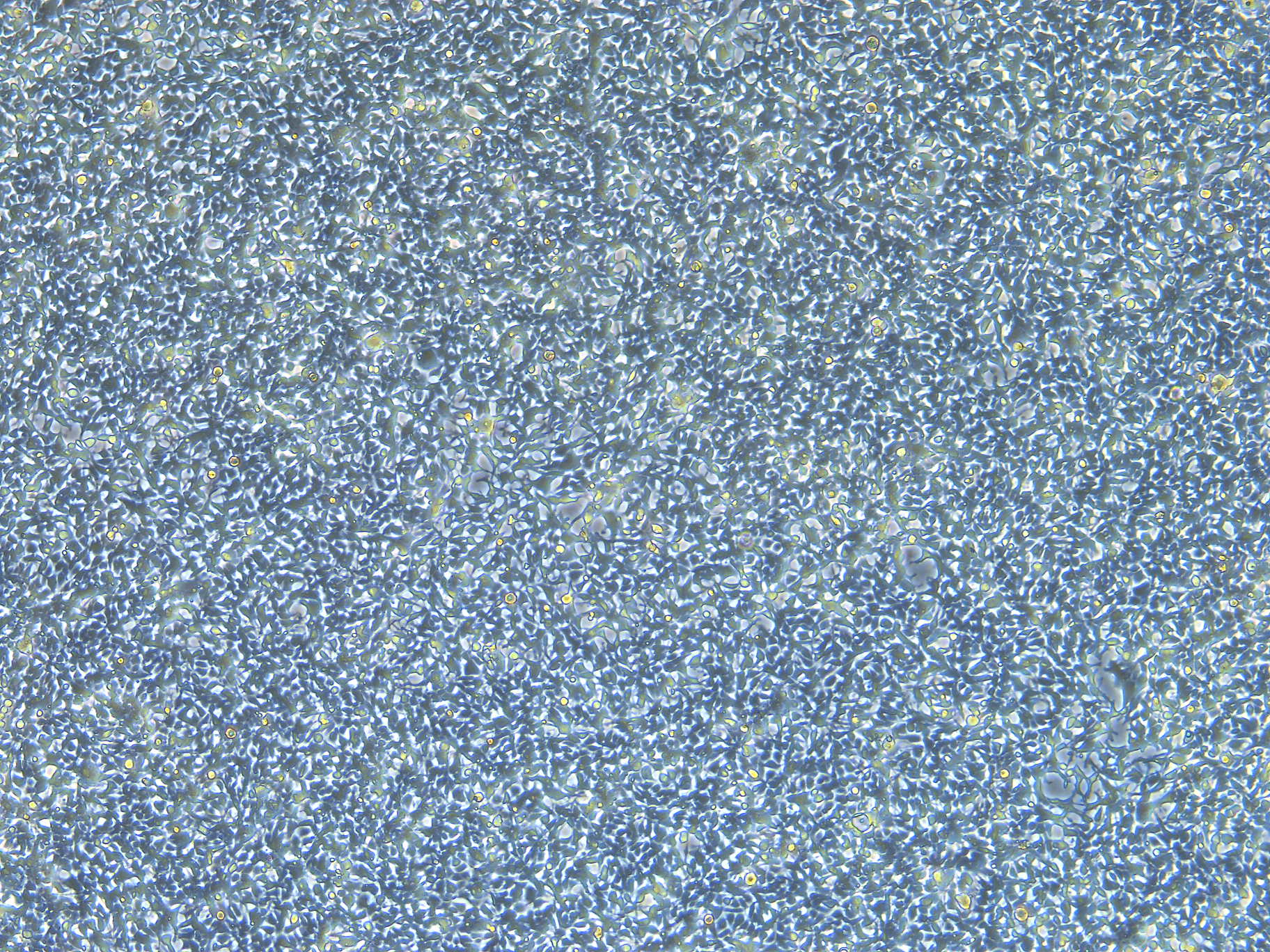

Supplement: Document S3. Microscopy raw files [file mmc3.zip › Microscopy/HEK293T-ACE2 Mock 48h.JPG]

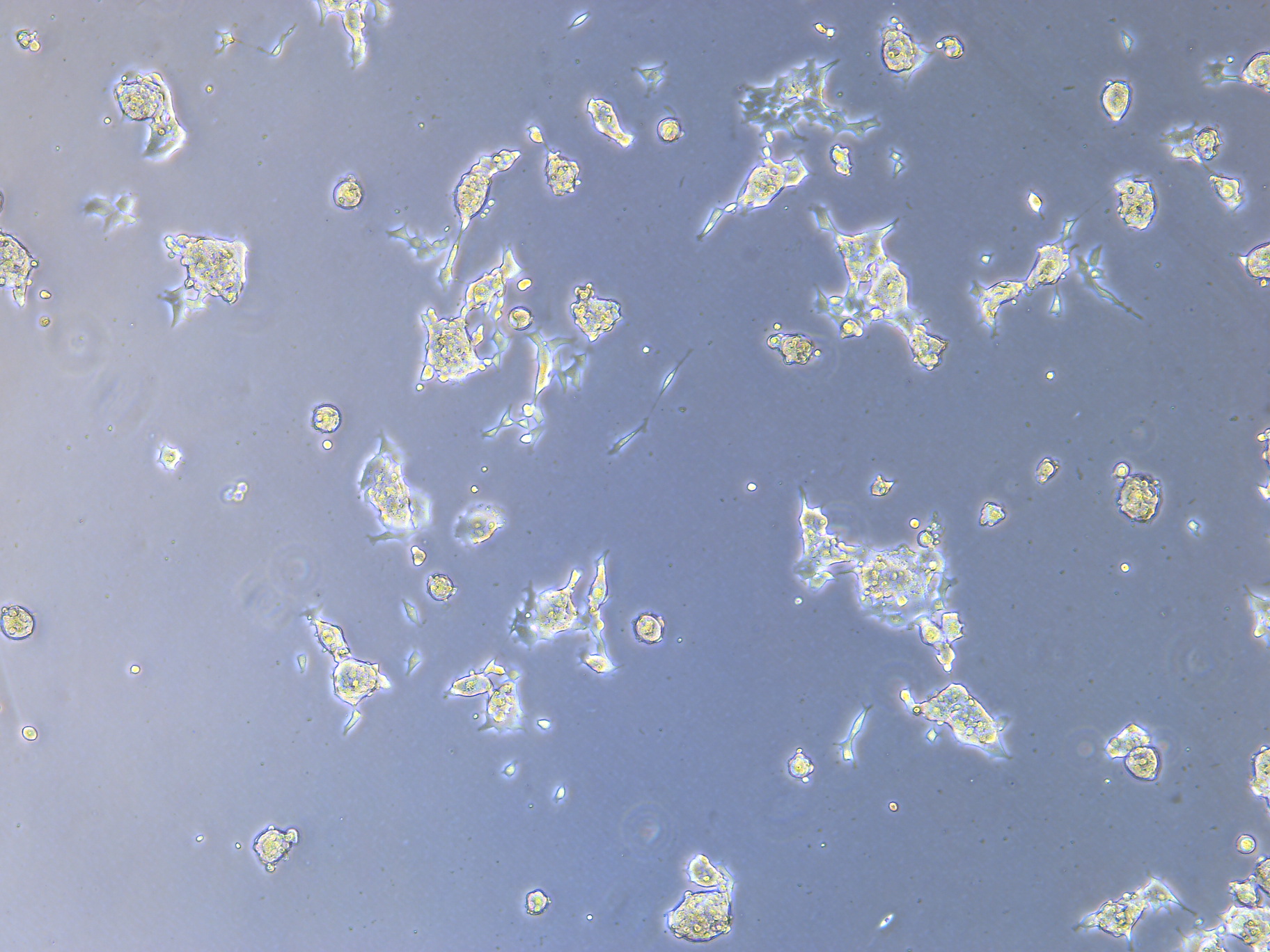

Supplement: Document S3. Microscopy raw files [file mmc3.zip › Microscopy/HEK293T-ACE2 SARS-CoV-2 24hpi.JPG]

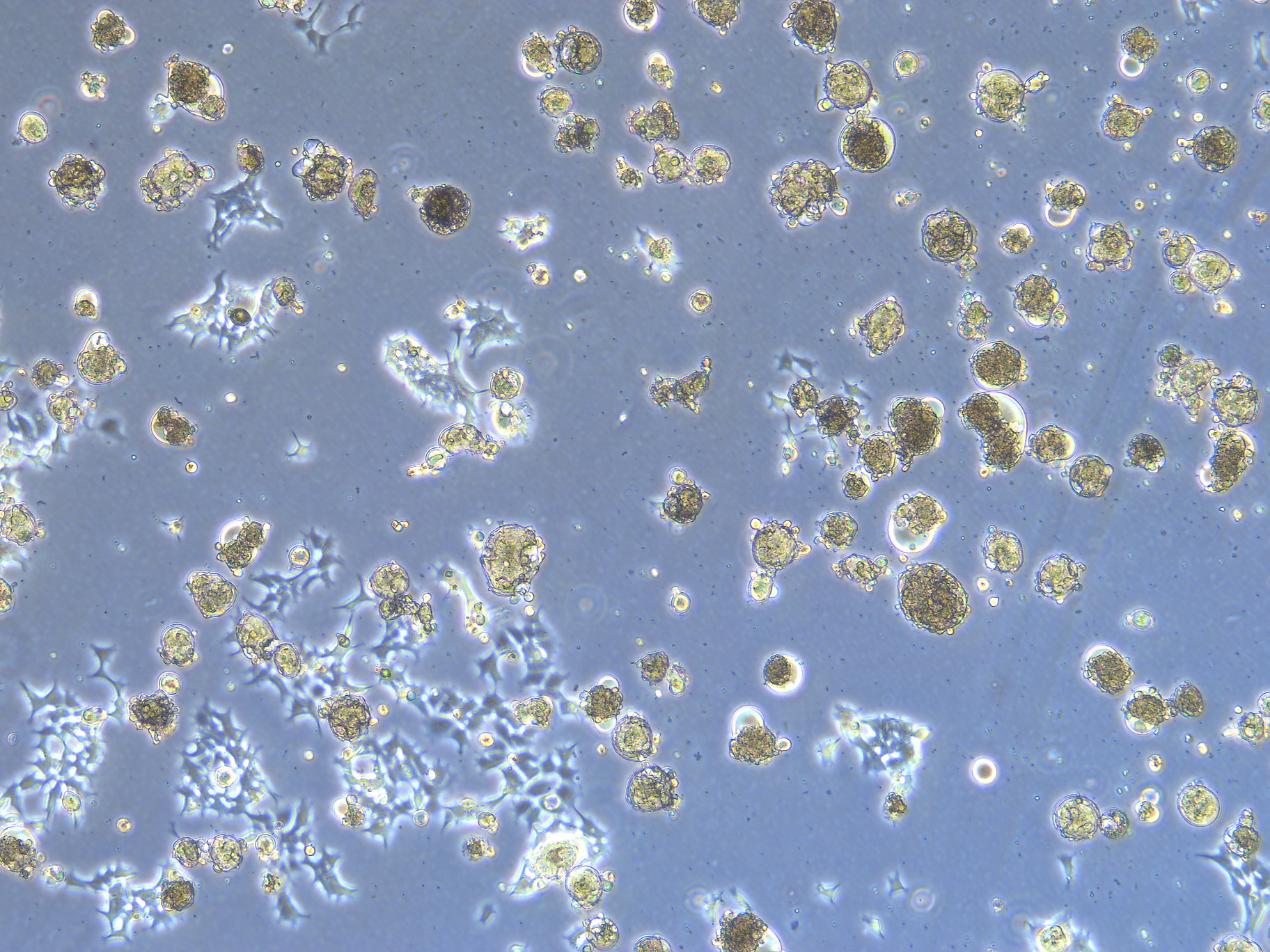

Supplement: Document S3. Microscopy raw files [file mmc3.zip › Microscopy/HEK293T-ACE2 SARS-CoV-2 48hpi.JPG]
